# Ruthenium Complexes with Pyridazine Carboxylic Acid: Synthesis, Characterization, and Anti-Biofilm Activity

**DOI:** 10.3390/molecules29235694

**Published:** 2024-12-02

**Authors:** Patrycja Rogala, Agnieszka Jabłońska-Wawrzycka, Grzegorz Czerwonka, Maciej Hodorowicz, Sławomir Michałkiewicz, Justyna Kalinowska-Tłuścik, Marta Karpiel, Katarzyna Gałczyńska

**Affiliations:** 1 Institute of Chemistry, Jan Kochanowski University, 7 Uniwersytecka Str., 25-406 Kielce, Poland; slawomir.michalkiewicz@ujk.edu.pl; 2 Institute of Biology, Jan Kochanowski University, 7 Uniwersytecka Str., 25-406 Kielce, Poland; gczerwonka@ujk.edu.pl (G.C.); katarzyna.galczynska@ujk.edu.pl (K.G.); 3 Faculty of Chemistry, Jagiellonian University, 2 Gronostajowa Str., 30-387 Cracow, Poland; hodorowm@chemia.uj.edu.pl (M.H.); kalinows@chemia.uj.edu.pl (J.K.-T.); marta.karpiel@doctoral.uj.edu.pl (M.K.); 4 Doctoral School of Exact and Natural Sciences, Jagiellonian University, 11 Lojasiewicza Str., 30-348 Cracow, Poland

**Keywords:** ruthenium complex, molecular structure, spectroscopy, microbiological test

## Abstract

As a result of drug resistance, many antimicrobial medicines become ineffective, making the infections more difficult to treat. Therefore, there is a need to develop new compounds with antibacterial activity. This role may be played, for example, by metal complexes with carboxylic acids. This study reports the formation and characterization of ruthenium complexes with pyridazine-3-carboxylic acid (pdz-3-COOH)—([(η^6^-*p*-cym)Ru^II^Cl(pdz-3-COO)] (**1**), [Ru^III^Cl_2_(pdz-3-COO)_2_Na(H_2_O)]*_n_*(H_2_O)_0.11_ (**2**) and [Ru^III^Cl_2_(pdz-3-COO)_2_Na(H_2_O)_2_]*_n_* (**3**). The synthesized compounds were analyzed using various spectroscopic and electrochemical techniques, with structure confirmation via SC-XRD analysis. Experimental data showed the ligand binds to metal ions bidentately through the nitrogen donor of the pyridazine ring and one carboxylate oxygen. To visualize intermolecular interactions, Hirshfeld surface analysis and 2D fingerprint plots were conducted. Furthermore, the impact of ruthenium compounds (**1** and **2**) on the planktonic growth of selected bacterial strains and the formation of *Pseudomonas aeruginosa* PAO1 biofilm was examined. Both complexes demonstrated comparable anti-biofilm activity and outperformed the free ligand. The effect of the complexes on selected virulence factors of *P. aeruginosa* PAO1 was also investigated. Compounds **1** and **2** show high suppressive activity in pyoverdine production, indicating that the virulence of the strain has been reduced. This inhibitory effect is similar to the inhibitory effect of ciprofloxacin. Within this context, the complexes exhibit promising antibacterial activities. Importantly, the compounds showed no cytotoxic effects on normal CHO-K1 cells. Additionally, a molecular docking approach and fluorescence spectroscopy were used to determine the interactions of ruthenium complexes with human serum albumin.

## 1. Introduction

Antibiotic resistance is currently one of the greatest threats to human health because antibiotics are no longer effective in treating the infectious diseases for which they were specifically designed. Therefore, there is an urgent need to develop new compounds with antibacterial properties. This work represents a continuation of our research on ruthenium complexes as potential anti-biofilm agents [1,2,3,4,5,6,7]. Here, we focused on the modification in the group of ruthenium complexes with heteroaromatic carboxylic acids. The reason for this modification is the moderate anti-biofilm activity of Ru complexes with imidazole, pyridine, and pyrazine derivatives [3,7]. The utilization of ruthenium complexes in this area seems justified, considering their previously described anticancer and antibacterial properties [2,3,4,5,6,7,8,9,10,11]. On the other hand, heteroaromatic carboxylates serve as an important class of ligands. So far, this type of ligand shows an interesting mode of coordination with Ru ions in various oxidation states caused by a strong affinity of the N and O donor atoms for the central ion. Unfortunately, the presence of the -COO group causes certain limitations related to transport through biological membranes. Modifications in the molecular structure of complexes, consisting of the use of proper ligands, make it possible to control biological properties, including features such as water solubility or the value of the potential for penetration through cell membranes. The designed model system should have such properties as low molecular weight or hydrolytic stability. A well-defined structure makes it possible to indicate the structural effects influencing biological activity. And this, in turn, allows us to confirm the possibility of the compound acting in terms of redox reactions or the ability of new metal complexes to interact with selected biological targets. Obtaining this information leads to the improvement of the model systems. Thus, our efforts are focused on improving the properties related to better solubility in water and greater permeability through biological membranes, and in effect better, anti-biofilm activity.

In this work, pyridazine-3-carboxylic acid (pdz-3-COOH), which is structurally similar to pyridine-3-carboxylic acid, commonly known as nicotinic acid, was employed as a model ligand. Pyridazinecarboxylic acid has also similar properties to used pyridinedicarboxylic acid although it is a stronger acid than the corresponding one. The structural similarity of both pyridazinecarboxylic and pyridinecarboxylic acids means that ligation with ruthenium ion for these ligands should occur in a similar N,O-manner. The presence of lone pairs of electrons on nitrogen and oxygen atoms means that pdz-3-COOH as a ligand can form bidentate compounds [12,13,14,15,16,17,18,19], bridged systems [20,21], and coordination polymers [22] with the central ions. Pyridazines are an important class of heterocycles that exhibit a wide range of biological actions, such as anticancer, antibacterial, antifungal, antiviral, anti-inflammatory, antihypertensive, and antidepressant activities [23,24,25,26,27]. For example, hydralazine is an antihypertensive agent used for the management of essential hypertension [28], minaprine is a psychotropic drug that has proved to be effective in the treatment of various depressive states [29], and cefozopran is a fourth-generation cephalosporin that has good activity against Gram-positive microorganisms [30]. As for the biological activity of pyridazine-3-carboxylic acid on selected strains of bacteria, fungi, and cancer cells, it was examined by Świderski’s team [31]. These studies showed that pdz-3-COOH exhibits weak or moderate biological activity. However, to the best of our knowledge, no previous study on the biological activity of metal complexes with pdz-3-COOH has been reported. Our aim is to attempt to synthesize carboxylate complexes, which would lead to the improvement of new and promising biological active agents. In this study, several dimensions are explored: (i) the synthesis of ruthenium complexes incorporating pdz-3-COO^−^; (ii) the determination of the crystal structures and physicochemical characterization of the synthesized ruthenium complexes; (iii) the investigation of the electrochemical properties of the Ru complexes; (iv) the evaluation of the antibacterial and anti-biofilm activities of Ru compounds against selected bacterial strains; (v) the assessment of the effects of Ru complexes on specific virulence factors. To enhance the development of effective antimicrobial agents, it is imperative to examine the potential for transport across biomembranes and the interactions with human serum albumin (HSA), which acts as a drug carrier. Drug–protein interactions were investigated using molecular docking, a predictive method that provides valuable information about the likelihood of the examined structures binding with HSA. Furthermore, the method of fluorescence quenching of human serum albumin was used as a complementary technique.

## 2. Results and Discussion

### 2.1. Preparation of the Complexes

**Figure 1:**
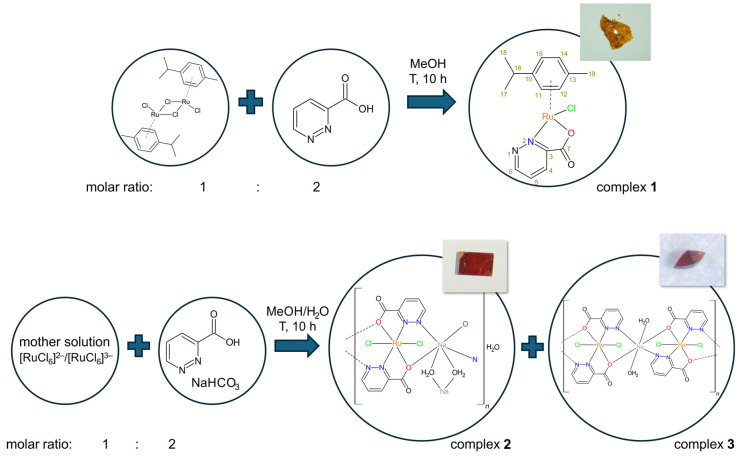
Synthetic route to the complexes ([(η^6^-*p*-cym)Ru^II^Cl(pdz-3-COO)] (**1**), [Ru^III^Cl_2_(pdz-3-COO)_2_Na(H_2_O)]*_n_*(H_2_O)_0.11_ (**2**), and [Ru^III^Cl_2_(pdz-3-COO)_2_Na(H_2_O)_2_]*_n_* (**3**).

Ruthenium complexes with pyridazine-3-carboxylic acid (pdz-3-COOH) were synthesized using two different sources of metal: a dimeric arene-ruthenium precursor ((η^6^-*p*-cym)Ru(μ-Cl)Cl]_2_) and 0.1 M ruthenium(III) chloride solution (mother solution). The treatment of the arene-ruthenium precursor with ligand in a molar ratio of 1:2 afforded ([(η^6^-*p*-cym)Ru^II^Cl(pdz-3-COO)] (**1**) in good yield. The Ru(III) complexes were successfully synthesized using the reflux method (at 65 °C) with the mother solution and a methanol-water solution of the ligand combined with sodium bicarbonate. The addition of NaHCO_3_ facilitated the deprotonation of the ligand. The Ru(III) ions coordinated with pdz-3-COO^−^ as well as Na^+^, which participated in the coordination to the same ligands, forming [Ru^III^Cl_2_(pdz-3-COO)_2_Na(H_2_O)]*_n_*(H_2_O)_0.11_ (**2**) and [Ru^III^Cl_2_(pdz-3-COO)_2_Na(H_2_O)_2_]*_n_* (**3**). Details related to the synthetic procedure used are presented in Figure 1. The main product is complex **2**. Meanwhile, complex **3** was obtained with a very low yield. Taking into account such low yield, it was not possible to perform biological studies for this complex. Furthermore, the characterization of complex **3** is limited to spectroscopic methods (IR, UV-Vis), electrochemical analysis, and a description of the crystal structure. The composition of the other complexes was established by elemental analysis, spectral methods, magnetic and electrochemical studies, and SC-XRD analysis.

### 2.2. Spectroscopic Characterization and Magnetic Measurements

The key infrared spectral bands indicating the coordination of pdz-3-COOH to central metal ions are summarized in Table S1 (ESI). A comparison of the IR bands of the free ligand and the metal complexes reveals significant differences (Figure S1, ESI). The free ligand displays a strong band at 1733 cm^−1^, corresponding to the νCOOH stretching vibration, while the complexes exhibit asymmetric (1676–1636 cm^−1^) and symmetric (1333–1304 cm^−1^) stretching bands of the carboxylate, confirming coordination to the Ru ion. The Δν value, calculated using the spectroscopic criterion established by Deacon and Phillips [32] (Δν = [ν(COO^−^)_asym_ − ν(COO^−^)_sym_]) and indicates a monodentate coordination mode of the carboxylate moiety in complex **1** (Δν = 324). For complexes **2** and **3**, Δν values exceeding 200 cm^−1^ suggest both monodentate and bridged coordination behavior of the carboxylate anion, resulting in polymeric structures consistent with crystallographic data. The infrared spectra of complexes **1**–**3** show bands in the 1587–1436 cm^−1^ range, attributed to νC=C, νC=N, and νN=N stretching vibrations in the pyridazine ring, which are shifted by 10–20 cm^−1^ relative to the free ligand. This shift indicates the involvement of nitrogen atoms in the aromatic ring of pdz-3-COOH in the formation of coordination bonds. Additionally, IR analysis identified water molecules (νO–H) above 3400 cm^−1^ and new peaks in the range from 670 to 540 cm^−1^ in the complexes. These peaks can be assigned to Ru–Cl vibrations based on standard samples RuCl_3_ and [(η^6^-*p*-cymene)Ru(µ-Cl)Cl]_2_.

The structure of the arene-ruthenium compound was analyzed using NMR spectroscopy. The ^1^H NMR spectrum shows a characteristic pattern for the *p*-cymene moiety: isopropyl protons appear as a doublet and septet, a singlet corresponds to the methyl group, and two doublets represent the arene ring protons. The aromatic region shows signals at 7.93 ppm (1H), 8.51 ppm (1H), and 9.54 ppm (1H), which are upfield shifted compared to the free ligand, indicating coordination with the ruthenium(II) ion. The absence of a signal for the COOH proton further supports the ligand’s coordination in its anionic form. The ^13^C NMR spectrum displays expected signals for coordinated *p*-cymene and pdz-3-COOH, with aromatic carbons of *p*-cymene between 79 and 101 ppm and the pyridazine ring at 125–155 ppm. Notable shifts, especially for C3 (Δδ_coord._ = +2.2), indicate ligand coordination. Furthermore, a singlet for the COO^−^ group at 170.24 ppm, shifted by +5.0 ppm, confirms the coordination of the carboxyl group with the metal ion.

The electronic absorption spectra of complexes **1**–**3** and pdz-3-COOH were recorded in distilled water (Figure S2, ESI), with UV-Vis data summarized in Table S2 (ESI). The analysis revealed intense intraligand π→π*/n→π* transition bands in the 200–310 nm range. Complex **1** also displayed a broad absorption peak at 386 nm, attributed to a metal-to-ligand charge transfer (MLCT) transition from the metal’s t_2g_ level to the ligand’s unoccupied π* molecular orbitals. The *d*–*d* transition bands in low-spin octahedral *d*^6^ complexes can be challenging to identify due to strong charge transfer bands [33] and are forbidden by the Laporte and spin selection rules. For complex **2**, broad absorption bands in the visible spectrum hindered the precise identification of the *d*–*d* transition peak. In the range of 320–540 nm, these bands can be attributed to a combination of ligand-to-metal charge transfer (LMCT: π(L)→d(Ru)) and *d*–*d* transitions. Similar observations were made for complex **3**, although a weakly intense band responsible for the *d*–*d* transition was identified at 474 nm. According to the Tanabe-Sugano diagram, this band corresponds to the ^2^T_2g_→^2^A_2g_,^2^T_1g_ transition (with the base term ^2^I for (t_2g_)^5^(e_g_)^0^). The UV-Vis studies also showed that the ruthenium complexes are stable in aqueous solution over a period of 24 h (time provided for biological experiments). The relevant bands were not shifted. Only slight changes in the intensity of the absorption maxima were observed (Figure S3, ESI).

Magnetic susceptibility measurement of complex **1** using Gouy’s method showed the diamagnetic nature of the compound. This confirms the divalent state of the ruthenium ion in the complex (*d*^6^ configuration). Complex **2** exhibited paramagnetic behavior at room temperature. The effective magnetic moment value of 2.24 μB is greater than the spin-only value anticipated for an octahedral system with one unpaired electron. Perhaps the reason is the polymeric structure of complex **2**. Due to the insufficient amount of complex **3** obtained, its effective magnetic moment could not be determined.

### 2.3. Molecular and Crystal Structure Description for Ru Complexes in Different Oxidation States

**Figure 2:**
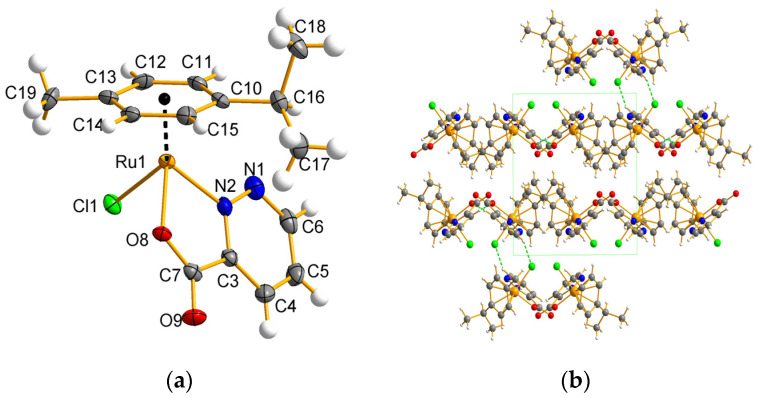
Molecular structure of complex **1** (**a**). Crystal packing with marked supramolecular interactions of C-H⋯Cl and C-H⋯O types forming *zig-zag* chains (view along *y*-axis) (**b**).

Single crystals of complex **1**, suitable for structure determination and obtained by slow evaporation of the methanolic solution, are crystallized in the *P*2_1_/c space group (ESI, Table S3). The molecular diagram of complex **1** is depicted in Figure 2, while Table 1 provides a comparative analysis of key bond distances and angles. The complex crystallizes as yellowish plates, containing a single neutral molecule per asymmetric unit. The molecular architecture reveals that the metal center is coordinated by an N,O-donor ligand and a terminal chloride, which occupy positions analogous to the legs of a piano stool, while the arene ring occupies the remaining coordination site, analogous to the seat of the piano stool (Figure 2).

Following deprotonation, pdz-3-COOH transitions from its carboxylic acid form to that of a carboxylate. The bidentate nature of the ligand facilitates the formation of a stable five-membered chelate ring with the ruthenium(II) ion. The plane of the ligand (pdz-3-COO) is oriented perpendicularly to that of *p*-cymene, presumably to minimize steric hindrance. The presence of strongly electronegative nitrogen atoms in close proximity results in the displacement of electron density away from the carbon atom to which the deprotonated carboxyl group is bonded, leading to a reduced electron density at this atom. Consequently, the C–C bond distances within the pyridazine ring exhibit inequivalence, with three shorter distances (N1–N2, N2–C3, and N1–C6) measuring approximately 1.32 to 1.36 Å, and three longer distances (C3–C4, C4–C5, and C5–C6) measuring around 1.38 to 1.40 Å, indicating concentrated electron localization around the nitrogen atoms within the ligand backbone. This finding contrasts with observations from our previous studies [2,7]. The geometry surrounding the ruthenium atom can be characterized as distorted tetrahedral, with the distance from the ruthenium ion to the *p*-cymene ligand measuring 1.6567(5) Å. The Ru–N and Ru–O bond lengths in complex **1** exhibit slight asymmetry, ranging from 2.053(5) to 2.083(4) Å (Table 1). Notably, these distances are shorter than those observed for other carboxylate ligands, suggesting a stronger coordination of the ligand to the central ion compared to other carboxylate ligands assessed by our research team. In contrast, the Ru–Cl bond displays a significantly longer distance of 2.4126(2) Å. The valence angles range from 78.10(2) to 133.09(1)°, demonstrating substantial deviations from the values expected for a regular polyhedron. The angle metrics are slightly more constrained than those observed in pyridine and benzimidazole derivatives. An alternative characterization of the coordination environment is the *pseudo*-octahedral three-legged piano stool structure.

The stability of the [(η^6^-*p*-cym)Ru^II^Cl(pdz-3-COO)] crystal lattice is further enhanced by C–H⋯Cl and C–H⋯O interactions. Analysis of the packing within the structure of complex **1** indicates that structural units are interconnected through C–H⋯O interactions, forming *zig-zag* chains (Figure 2b), with the C–H group of the pyridazine ring participating in these interactions. The stability of these chains is reinforced by C–H⋯Cl interactions.

**Figure 3:**
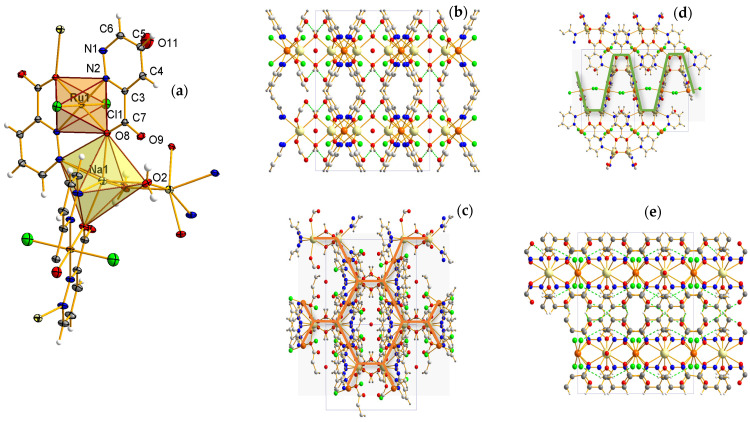
Molecular structure of compound **2**, including the numbering scheme and coordination polyhedra (**a**). Crystal network with intramolecular hydrogen bonds of the O–H⋯O type highlighted, and a channel filled with water molecules (view along the *z*-axis) (**b**). The two-dimensional layer formed by coordinative chains exhibiting a honeycomb topology (view in the *y*^*z* plane) (**c**). *Zig-zag* chains composed of ruthenium and sodium positions (view in the *x*^*y* plane) (**d**). Crystal packing with marked supramolecular interactions of the C–H⋯O and C–H⋯Cl types (view along the *x*-axis) (**e**).

The asymmetric part of the unit cell of the structure of compound **2** consists of the Ru(III) cation (occupancy 0.5, inversion center) and its coordinating bidentate pyridazinecarboxylate ligand and chloride anion. The composition is completed by a Na^+^ cation (occupancy 0.5) and two water molecules of which O2 plays the coordinating role, while O11 is the water of crystallization. The coordination environment of the Ru(III) cation adopts the geometry of a slightly distorted octahedron with a small axial deformation (Figure 3a).

Two symmetrically dependent pyridazinecarboxylate chelating ligands coordinate the ruthenium cation in a transplanar arrangement via N(2) and O(8) atoms, while two chloride ions (Cl(1) and Cl(1) according to the 3/2–x,3/2–y,1–z symmetry) occupy axial positions. The resulting 5-membered chelate ring is nearly planar with distortion angles of 1.65°. The remaining ruthenium complexes (**1** and **3**) tend to have a similar coordination bond mode. The bond lengths of Ru–N and Ru–O (2.030(1) and 2.033(1) Å) are found to be larger than Ru–Cl (2.335(4) Å) due to the Jahn–Teller effect. The polyhedron distortion is also caused by the N–Ru–O angles (Table 2) depending on the ligand structural parameters. It is observed that in the -COO^−^ group the C7–O8/O9 bonds are strongly asymmetric (more so than in complex **1**). This is probably the result of the concentrated electron localization in the region of the nitrogen atoms in the ligand skeleton. As in complex **1**, the C–C bond distances (for the pyridazine ring) become more nonequivalent, three of them N(1)–N(2), N(2)–C(3) and N(1)–C(6) are short (~1.34 Å), and three C(3)–C(4), C(4)–C(5) and C(5)–C(6) are long (~1.38 and 1.39 Å). The Na^+^ cation present in the structure is coordinated by two nitrogen atoms N(1) of the pyridazinecarboxylate ligand, two symmetrically dependent water molecules O2 and two carboxyl oxygen atoms O(8) (Figure 3a). Its coordination environment adopts a distorted octahedral geometry, and the Na–O and Na–N bond lengths are typical for this type of system. The water molecule O(2) forms a water bridge to the symmetrically dependent Na^+^ cation [1–x, 1/2–y,z]. It is noteworthy that the carboxyl oxygen atom O(8) simultaneously coordinates two different cations, Ru^3+^ and Na^+^, at distances of 2.033 Å and 2.490 Å, respectively. A perspective view of the hydrogen bonding network in complex **2** is shown in Figure 3b. The bond lengths and angles observed for the hydrogen bond interactions are given in Table S4 (ESI).

In the crystal structure of compound **2**, there are three types of hydrogen bonds that stabilize the structure. The observed strong interactions involving the C(4) and C(5) atoms of the pyridazine ring and the adjacent O(9) atoms have D⋯A lengths of 3.131(1) and 3.362(1) Å, respectively. The relatively weak interaction between the pyridazine C(6) atom and the chloride ion Cl(1) has a D⋯A length of 3.614(1) Å. The Ru complexes are connected by sodium cations acting as linkers, forming coordinative chains in the *z*-axis (Figure 3d). The individual chains are linked together by double water bridges Na^+^⋯H_2_O⋯Na^+^, whose formation involves water molecules O(2). The two-dimensional layer composed of these coordinative chains exhibits a honeycomb topology (Figure 3c).

**Figure 4:**
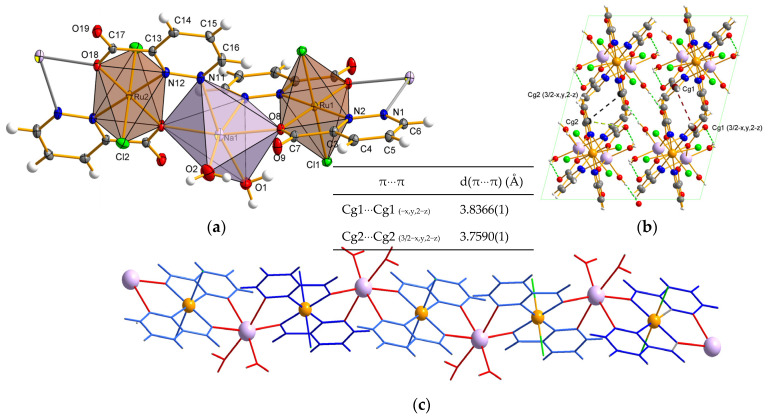
Molecular structure of **3** with numbering scheme and coordination polyhedra (**a**). The infinitive chain built from Ru units and Na units (view along *y*-axis) (**b**). 3-dimensional network with marked supramolecular interaction (**c**). (Cg1 denotes center of gravity of N1–N2–C3–C4–C5–C6 ring (pyridazine); Cg2 denotes center of gravity of N11–N12–C13–C14–C15–C16 ring (pyridazine)).

The crystal structure of **3** is shown in Figure 4a. The polymeric complex crystallizes into a monoclinic system with the *I* 2/a space group. The monomeric unit is formed by three metal centers: two ruthenium and one sodium. Each ruthenium(III) ion coordinates with two carboxylates in N,O-chelating manner (by carboxyl oxygen atoms (O8) and two nitrogen atoms (N2) originating from two pzd-3-COO ligands). Coordination sphere is completed with two chloride anions, forming an elongated octahedron. This phenomenon can be attributed to the Jahn-Teller effect. Selected bond lengths and angles are given in Table 3. The axial positions are occupied by two Cl^−^ ions with bond lengths of 2.3402(4) and 2.3285(5) Å (Table 3). The equatorial plane is formed by two *trans*-related N,O-bidentate pyridazine-3-carboxylate ligands. The Ru–N and Ru–O bonds are nearly symmetrical, similar to those in complex **2**, with bond lengths ranging from 2.0311(13) to 2.0388(1) Å. In general, distortion of polyhedron is caused by Ru-Cl bond lengths as well as values of valence angles, of 80 and 92.55°. The same four ligands are engaged in coordination with the sodium ion through N1, N11, O8, and O18 atoms.

As is shown in Figure 4, two N donors from the same pzd-3-COO coordinate with two different metal centers from the sides of the pyrazine ring. To the best of our knowledge, this is the only case of pyridazine monocarboxylates known in the literature in which a tridentate coordination mode is observed (for complex **2** and **3**). In the Na surrounding, two pdz-3-COO are arranged in the *cis* position relative to each other. Two other coordination sites of the sodium are completed by water molecules (O1 and O2). Carboxylates act as monodentate bridging ligands. Such binding modes between metal centers offer unique flexibility. Despite of this, the –C3–C7–O8–O9 fragment displays planarity. Lippard et al. [34] proposed that the monodentate bridging mode serves as a significant intermediate between more prevalent modes of carboxylate attachment. Notably, complexes featuring monodentate bridging carboxylates typically include metal ions in relatively low oxidation states. The literature lacks instances of carboxylates using a single oxygen atom to bridge two metal centers with oxidation states exceeding +II, despite the existence of higher redox potentials. As one would expect, the carboxylate contribution in M–O(carboxylate) bonds is inequivalent, as it occurs significant disproportion. The Na–O bond is longer than the Ru–O definitely (Table 3). Between two different metal centers a 5-membered ring is formed. As a geometry of the sodium coordination sphere is very distorted, it can be described as an octahedron or trigonal antiprism. The Na–O and Na–N bonds lengths vary and are in the scope of 2.2996(2) to 2.5726(2) Å. They are in good agreement with those reported in the literature [35,36]. However, the Na–O bonds are slightly shorter than those in complex **2**, while the Na–N bonds are significantly longer.

Analysis of crystal packing revealed that the ruthenium units {RuL_2_Cl_2_} are connected (with dihedral angle of 59.54°) through the sodium ion, forming an infinitive chain (Figure 4c) that extends into 1D space. There are intermolecular strong hydrogen-bonding interactions of the O–H⋯O and O–H⋯Cl type between water molecules and pzd-3-COO^−^ or chloride ions from adjacent Ru-units (Figure 4b). All these interactions promote the stability of the infinitive chain. Further stabilization of crystal lattice arises via a lot of supramolecular interactions of the C–H⋯O, C–H⋯Cl, and C–O⋯π type between pyridazine or carboxylate and the water, chloride anions and -COO^−^ groups or aromatic ring, respectively. The π-π stacking interactions between the pyridazine rings ensure that adjacent chains are joined together. The interactions between rings are quite close and have a shift of 1.603 Å. Finally, the chain arrangement is connected together in the extensive 3-dimensional network with rhombic motifs (Figure 4b). The geometry of the hydrogen bonds is summarized in Table S4 (ESI).

### 2.4. Hirshfeld Surface Analysis (HS)

**Figure 5:**
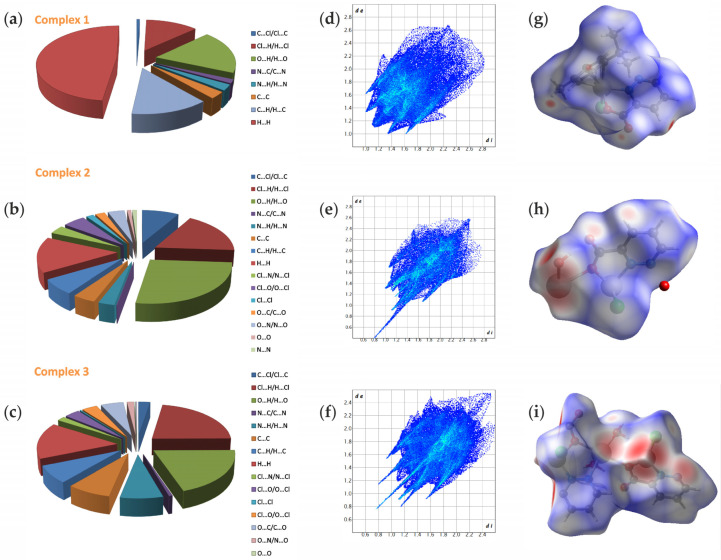
Relative percentage contributions of close contacts to the Hirshfeld surface in Ru complexes (**a**–**c**). The 2D fingerprint plots of all intermolecular interactions for Ru complexes (**d**–**f**) with percentage of interaction. The Hirschfeld surfaces highlight the relevant *d*_norm_ surface patches associated with the specific contacts for ruthenium complexes (**g**–**i**).

To achieve a more thorough understanding of the supramolecular structure of the compounds in question, we present an analysis of the Hirshfeld surface. This methodology serves as an effective tool for exploring both classical and unconventional types of intermolecular interactions. The visualization of the three-dimensional Hirshfeld surface (*d_norm_*) mapped onto the asymmetric unit of the studied compounds is depicted in Figure 5g–i. The intense red regions on the surface, colored according to *d_norm_*, correspond to interactions involving chlorine, nitrogen, and oxygen atoms, which align with the hydrogen bonds detailed in Table S4. In contrast, blue regions indicate longer contacts with a positive *d_norm_* value, while white regions represent distances of contacts that are exactly equal to the van der Waals separation, corresponding to a *d_norm_* value of zero. Figure 5d–f presents the associated fingerprint plots for complexes **1**, **2**, and **3**, revealing the significant contributions of various intermolecular interactions represented on the Hirshfeld surface. The fingerprint plots can be decomposed to emphasize specific atom–pair close contacts.

Overall, the largest contribution to the total Hirshfeld surface is attributed to H⋯H interactions, which comprise 49.3% in complex **1**, largely due to the abundance of hydrogen atoms on the molecular surface. Other notable interactions include O⋯H/H⋯O contacts, contributing 19%. H⋯C/Cl⋯H and C⋯H/H⋯C contacts represent the third most frequent interactions, accounting for 13.7% and 12.7%, respectively. The crystal structure indeed features C–H⋯Cl hydrogen bonds. Other interaction types depicted in Figure 5a contribute less than 3% of the total contacts. Consequently, this analysis indicates that H⋯H contacts are the primary driving force behind molecular arrangement and crystal packing. In contrast, the polymeric structure of complex **2**, while sharing the same ligand, exhibits a different distribution of contact contributions to the total Hirshfeld surface. In complex **2**, the predominant contribution (20.3%) arises from reciprocal H⋯O contacts. These interactions also manifest in the fingerprint plot as an inner wing and the sharpest asymmetrical spike, indicating the close proximity of H⋯O contacts within the crystal packing. These interactions are associated with the presence of hydrogen bonds of the type O–H⋯O, as well as weaker interactions of the type C–H⋯O. Contributions from Cl⋯H/H⋯Cl (13.4%) and H⋯H (11.4%) interactions also significantly contribute to the total surface area. Smaller contributions to the Hirshfeld surface area are attributed to C⋯H/H⋯C contacts, which account for 5.0% and are represented by a wide area with low distribution. Strikingly, C⋯C interactions account for only 3.1% of the total area. This suggests that hydrogen bonding and van der Waals interactions play crucial roles in the crystal packing of complex **2**. For complex **3**, the decomposition of fingerprint plots indicates that reciprocal H⋯Cl and O⋯H contacts, respectively comprise 20.6% and 18.4% of the total Hirshfeld surface area (Figure 5i), representing the most prevalent interactions. The abundance of C–H⋯Cl, O–H⋯Cl, C–H⋯O, and O–H⋯O type hydrogen bonds contributes to this prevalence. The reciprocal H⋯Cl contacts appear as two outer spikes, suggesting significant van der Waals interactions between chloride and hydrogen atoms in the crystal lattice. Two sharp inner spikes representing O⋯H/H⋯O contacts indicate strong hydrogen bond interactions involving O1–H1B⋯O19 and O2–H2B⋯O9. Hydrogen bonds involving oxygen and hydrogen atoms are known to be highly directional, significantly impacting the structural arrangement of molecules within a crystal lattice. The H⋯H interactions are the second most frequent (14%) (Figure 5c), reflecting the substantial hydrogen presence on the molecular surface. C⋯H/H⋯C contacts account for 7% (Figure 5c) of the Hirshfeld area, with a similar contribution observed for C⋯C contacts (7.4%). A π⋯π stacking interaction is evidenced by the presence of alternating triangular regions of red and blue surrounding the aromatic rings on the shape index surface for complex **3**. These aromatic stacking interactions play a vital role in the supramolecular arrangements of this compound and contribute to hydrophobic interactions with proteins. All other observed interactions contributed less than 3.5%. Figure S4 (ESI) illustrates the comparison of the percentages of common intermolecular contacts occurring across all investigated ruthenium complexes.

### 2.5. Electrochemical Studies

**Figure 6:**
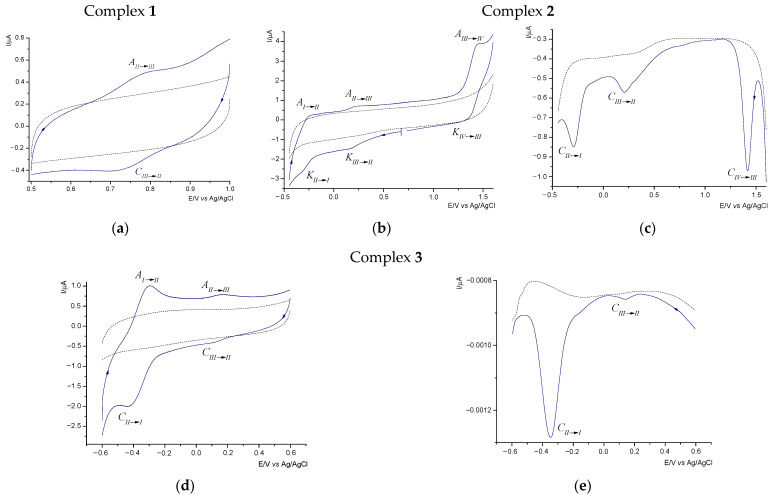
CV (**a**,**b**,**d**) and DPV (**c**,**e**) curves recorded in acetonitrile-ethanol mixture containing 0.1 M TBAPF_6_ and 1 mM Ru(II) complex/Ru(IV) complex/Ru(III) complex (—) or 1 mM ligands (---), (CV conditions: GCE, Ø = 2 mm, scan rate 100 mV s^−1^, T = 25 °C; DPV conditions: CF, Ø = 33 µm, pulse amplitude 20 mV, pulse width 80 ms, scan rate 20 mV s^−1^).

Cyclic voltammetry (CV) and differential pulse voltammetry (DPV) are widely utilized electrochemical techniques that provide both qualitative and quantitative insights into electron transfer mechanisms, the reversibility of electrode processes, and the formal redox potential. The CV curves for complexes **1**–**3** were recorded on a glassy carbon (GC) electrode within the potential ranges of 0.50 to 1.00 V for complex **1**, 1.50 to −0.50 V for complex **2**, and 0.6 to −0.6 V for complex **3**, referenced against Ag/AgCl. These measurements were conducted at scan rates of 50, 100, 200, and 500 mV s^−1^. The complexes were dissolved in a mixture of acetonitrile and ethanol (*v*:*v*, 3:2) containing 0.1 M tetrabutylammonium hexafluorophosphate (TBAPF_6_) as the supporting electrolyte. Representative voltammograms are depicted in Figure 6, while Table 4 summarizes the electrochemical data for the complexes analyzed. To assess the reversibility of the redox couples and to determine the number of electrons transferred (n), we applied the CV diagnostic criterion, Δ*E*_p_ = *E*_pa_ − *E*_pc_ (where *E*_pa_ and *E*_pc_ represent the anodic and cathodic peak potentials, respectively) applied [37]. An additional criterion confirming the reversibility of the electrode process and stability of the reaction primary product is the ratio of the reverse and forward peak currents being close to unity. It is noteworthy that the ligand does not exhibit electroactivity under the experimental conditions, as indicated by the dashed line in Figure 6.

The voltammogram of complex **1** shows only one redox pair. Both anodic and cathodic signals are poorly shaped peaks (Figure 6a). As we have established, this redox pair corresponds to the Ru(II)↔Ru(III) oxidation/reduction process. The Ru-centered oxidation observed in the potential range of 0.70 to 0.90 V vs. Ag/AgCl is also reported for analogous *p*-cymene complexes of Ru(II) [6,7]. In the case of complex **1**, we found that as the scan rate increased, the peak-to-peak separation (Δ*E*_p_) exhibited only a minor increase (Table 4). This behavior suggests that the electrode process is *quasi*-reversible. By applying the criterion Δ*E*_p_ = 0.058/n V, we can conclude that the process involves the transfer of a single electron. This is evidenced by Δ*E*_p_ close to the theoretical value of 0.058 V especially at low potential scan rates.

As many as three redox pairs have been identified for complex **2**, which contains a metal ion in the +III oxidation state. As shown in Figure 6b, the cathodic process began at a potential of 0.75 V, with the first electrode polarization occurring towards negative potentials. The first peak observed at 0.160 V is attributed to the reduction of Ru(III) to Ru(II). The second peak at −0.310 V is related to the subsequent reduction of Ru(II) to Ru(I). In the anodic cycle of the CV curve (Figure 6b), the signals within the potential range of −0.20 to 0.25 V are assigned to the oxidation processes of Ru(I) to Ru(II) and Ru(II) to Ru(III) (Table 4). Additionally, a further anodic peak is noted on the curve near 1.45 V, which represents the transition from Ru(III) to Ru(IV). This peak is linked to the cathodic signal at 1.350 V, corresponding to the reduction of Ru(IV) back to Ru(III). Similar observations were made for the ruthenium(III) complex with 2-(2′-pyridyl)benzimidazole, as described in [1]. For the redox couple Ru(IV)↔Ru(III), the CV diagnostic parameters exceeded the theoretical value of 0.058 V, which is expected for a reversible one-electron redox couple, indicating that this redox system is *quasi*-reversible. This conclusion is further supported by the gradual increase of *E*_pc_ and *E*_pa_ values with rising scan rates. For the other two redox pairs (Ru(III)↔Ru(II) and Ru(II)↔Ru(I)), both cathodic and anodic signals show slight shifts as the scan rate increases. The *quasi*-reversible nature of the electrode reactions is evidenced by minor changes in the Δ*E*_p_ value with increasing scan rates, which remain close to the theoretically expected value (0.058 V) predicted for the exchange of a single electron. The presence of three distinct redox systems is also confirmed by the DPV curve. Analysis of the DPV curve (Figure 6c) revealed the presence of three distinct maxima, which are attributed to the reduction of ruthenium in the +IV, +III, and +II oxidation states.

The redox behavior of complex **3** was examined and is presented in Figure 6d. There are two cathodic peaks at potentials of 0.11 and −0.43 V (at a scan rate of 100 mV/s); the first one is rather poorly shaped, while the current of the second cathodic peak is much higher. The first signal corresponds to the anodic peak on the oxidation curve, appearing at a value of 0.17 V. This redox pair is related to the reduction/oxidation of Ru(III)-Ru(II). The peak-to-peak separation value (Δ*E*_p_) recorded for different scan rates indicates the *quasi*-reversible nature of this redox pair (the cathodic and anodic signals are slightly shifted with the increase of the scan rate), and one electron participates in the described process (the CV diagnostic criteria are close to the theoretical value of 0.058 V) (Table 4). In turn, the cathodic peak at −0.43 V, together with the anodic peak observed at −0.29 V (at a scan rate of 100 mV/s), produces a second redox system in which the reduction/oxidation reaction of Ru(II)-Ru(I) occurs. Here, the Δ*E*_p_ value is 0.14, which demonstrates the irreversible nature of the electrochemical process. This is confirmed by significant shifts of the anodic and cathodic peaks with increasing scan rates (Table 4). The applied CV criteria prove a single-electron transfer. As can be seen in Figure 6e, the DPV curve confirms the presence of two distinct maxima, which are assigned to the ruthenium reduction in the +III and +II oxidation states.

### 2.6. Minimum Inhibitory Concentration and Biofilm Biomass Quantification

**Figure 7:**
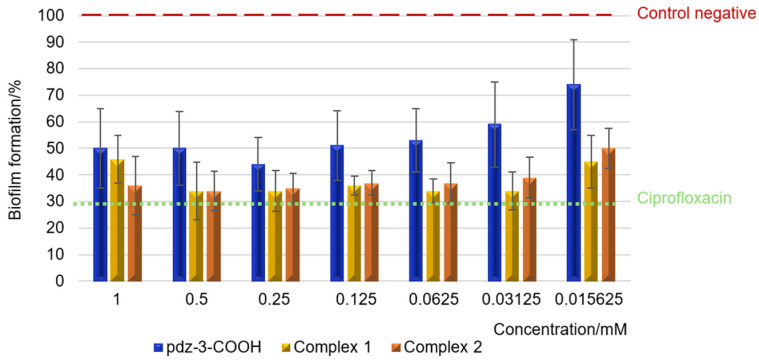
*P. aeruginosa* PAO1 biofilm formation in the presence of ligand, complex **1**, complex **2**, and ciprofloxacin. The absorbance of the negative control was considered to represent 100% of biofilm formation (data are presented as mean ± SD, *n* = 4).

To evaluate the bacteriostatic properties of the ligand and the ruthenium complexes (**1** and **2**) against *Escherichia coli*, *Staphylococcus aureus,* and *Pseudomonas aeruginosa*, MIC values were determined. In addition to the metal complexes, the MIC of the positive control (ciprofloxacin) and starting materials (metal salt, ruthenium precursor) was also determined. The MIC value was expressed as the lowest concentration to inhibit the visible growth (no turbidity observed) of the test strains following 24 h incubation at 37 °C. The results are summarized in Table S5. The analysis of the results showed that the starting compounds used for the synthesis of metal complexes do not reveal any remarkable influence on the growth of the tested bacteria. In turn, complexes **1** and **2** have antibacterial activity at the highest tested concentration (1 mM) against all three bacteria strains used in the studies. The MIC values in this study are comparable to results for ruthenium complexes with heteroaromatic carboxylic acids reported in our previous paper [3]. Ciprofloxacin inhibits bacterial growth at concentration lower than 62.5 μg/mL. Although bacteria are single-celled organisms, in natural and clinical settings they occur mainly in biofilms. To assess the ability of the tested complexes to prevent the *P. aeruginosa* PAO1 biofilm formation, bacterial cells were pre-treated with the ruthenium complexes or ciprofloxacin for 24 h. The biomass of biofilm formed was examined by crystal violet staining assay. The results of the biological activity of the compounds against the *P. aeruginosa* PAO1 are presented in Figure 7.

Inhibition of *Pseudomonas aeruginosa* PAO1 biofilm formation in the presence of ruthenium complexes occurs at concentrations lower than those required to restrict planktonic bacterial growth, as previously outlined. Biofilm formation inhibition assay shows that even the lowest concentrations of the tested complexes **1** and **2** significantly affect the amount of biofilm, expressed as the absorption of crystal violet released from the cells settled in the biofilm. Additionally, it was observed that the biological activity of complexes **1** and **2** is comparable. The biomass of biofilm is inhibited 66% by complexes **1** and **2** at a concentration of 0.5 mM. A similar reduction effect was obtained for a concentration of 0.25 mM—64% (**1**) and 66% (**2**), and for a concentration of 0.125 mM—63% (**1**) and 64% (**2**). It is worth noting that these values are similar to those obtained for the reference standard being evaluated—ciprofloxacin. Interestingly, it should be noted that the highest and the lowest concentrations of the ruthenium compounds used (1 mM and 0.015625 mM) have slightly less anti-biofilm effect than the tested concentrations in the range from 0.03125 to 0.5 mM (Figure 7). In the case of the Ru(II) complex, increasing the concentration of the added compound (1 mM) resulted in a reduction of the biofilm biomass by 55% at the tested concentration. Moreover, in this test, the ligand shows the weakest ability to inhibit biofilm formation. It is also worth mentioning that the activity of the Ru(IV) complex with pyridazine-3-dicarboxylic acid against the *P. aeruginosa* PAO1 biofilm at a concentration of 1 mM, described in this work, is almost identical to the activity of the Ru(IV) complex with pyridine-2,3-dicarboxylic acid (compound previously obtained by our group). This time it turned out that the modification of the structure of the Ru(IV) complex (another heteroaromatic acid used) did not affect the higher activity of the compound (taking into account the concentrations of 1 mM compounds).

### 2.7. Pyoverdine Inhibition Assay

**Figure 8:**
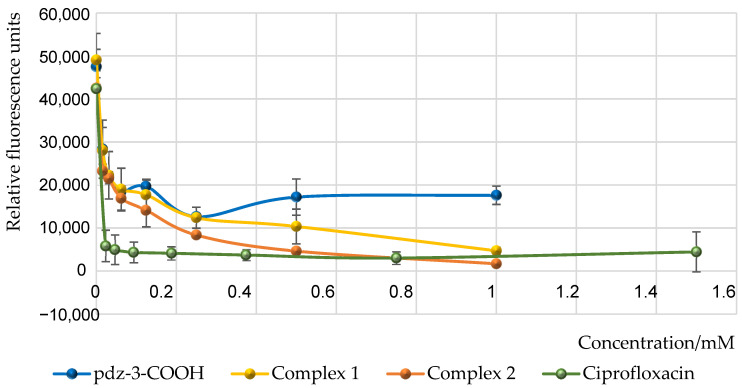
Level of pyoverdine secretion by *P. aeruginosa* PAO1 strain after incubation with the complexes **1** and **2**, and ciprofloxacin as a positive control.

*Pseudomonas aeruginosa* causes many serious and acute infections due to its wide variety of virulence factors. These factors contribute to the development of multidrug-resistant strains, which are a major reason for the low effectiveness of therapeutic drugs. The siderophore pyoverdine is considered one of the most important virulence factors of this pathogen because it chelates iron ions from the environment and transports them into the bacterial cell during infection [38]. It acts as a nutrient essential for growth. In addition to its role in nutrition, pyoverdine is implicated in biofilm formation and cell-to-cell communication [39]. It is produced in response to a signal from a *quorum sensing* (QS) system using homoserine lactones as an autoinducer molecule. Considering how virulence factors play a role in the pathogenesis of infections, the regulation of their production is extremely important. Thanks to the phenomenon of fluorescence, pyoverdine can be easily detected in the environment and quantitatively measured by exciting it at an excitation wavelength of λ = 398 nm and observing the emitted light at an emission wavelength of λ = 455 nm. Our experiment aimed to assess the effect of the ruthenium complexes on the production capacity of pyoverdine by *P. aeruginosa* PAO1 at various concentrations. The effect of the applied compounds on the secretion of pyoverdine is shown in Figure 8 (data are expressed as the relative light units (RLU)).

The observed reduction in pyoverdine production clearly evidences that the virulence of the *P. aeruginosa* PAO1 strain decreases in the presence of the tested compounds. It was found that pdz-3-COOH at the highest concentration tested causes noticeable, albeit the smallest among the studied compounds, reduction in pyoverdine production (by 61%). The ruthenium compounds at a concentration of 1 mM were able to inhibit pyoverdine production by 90% (**1**) and 96% (**2**) compared to the negative control (TSB medium with inoculated bacteria). This inhibitory effect of the complexes is comparable to that of the control agent—ciprofloxacin. Importantly, the inhibition of pyoverdine by the complexes is also visible at concentrations of 0.03125 mM, where the fluorescence is reduced by more than 50%. The effectiveness of the compounds is probably due to changes in the iron uptake system and modulation of signaling pathways, resulting in a reduction in the ability of bacteria to survive.

### 2.8. Cytotoxicity Activity

Cytotoxicity studies are important when characterizing new compounds because they allow for the assessment of the toxicity of the test substance. Minimal or no toxicity to normal cells is essential for the successful development of a biologically active preparation. Metabolic viability assay of the Chinese hamster ovary (CHO-K1) and the adenocarcinoma human alveolar basal epithelial (A549) line cells treated with ruthenium complexes was performed using the MTS assay. By utilizing both CHO-K1 and A549 cell lines in cytotoxicity testing, we aimed to obtain a comprehensive assessment of potential toxic effects in different cell types, leading to more robust conclusions regarding the safety and efficacy of new compounds. Cytotoxicity tests of complexes **1** and **2** showed that in the tested concentration ranges, they do not inhibit the metabolic activity of normal CHO-K1 and cancer A549 cells (Figure S5, ESI).

### 2.9. HSA Binding Studies

To identify a promising candidate compound, it is essential to collect data regarding its ADMET properties—absorption, distribution, metabolism, excretion, and toxicity [40]. Drugs are primarily distributed in the bloodstream through reversible binding to plasma proteins, which serve as carriers. Serum albumin, a significant protein in circulation, is capable of transporting a wide range of compounds, including both small molecules and macromolecules. Its role as a reservoir for chemotherapeutic agents can enhance bioavailability [41]. The binding of a drug to protein must be sufficiently strong to facilitate transport while remaining weak enough to allow for the release of the drug at the biological target, thereby eliciting a therapeutic effect. Generally, two distinct areas are recognized as binding sites. Ligands that are tightly bound to site 1 typically fall into the category of dicarboxylic acids and/or bulky heterocyclic molecules, characterized by a negatively charged group localized at the center of the molecule. In contrast, site 2, which comprises a largely apolar cavity, is capable of binding aromatic carboxylic acids that feature a negatively charged acidic group at one end of the molecule [42].

To predict ligand–albumin interactions we applied molecular docking studies and fluorescence spectroscopy as complementary techniques (FS). The molecular docking method allows for revealing the binding site and the binding mode between HSA and the Ru-complex. In turn, the FS method gathers data and allows for assessing quantitatively binding strength between them. The Stern–Volmer (SV) and double logarithm Stern–Volmer (DLSV) equations establish a connection between fluorescence quenching phenomena and binding parameters, specifically the Stern–Volmer constant (K_sv_). This relationship holds true provided that albumin is the sole fluorophore present in the solution, that only a single type of interaction occurs, and that the concentration of the unbound agent significantly exceeds that of the bound fraction [43].

#### 2.9.1. Molecular Docking of the Studied Ruthenium Complexes to HSA

Initially, to find the most suitable HSA regions with the highest affinity towards complex **1**, the docking simulations included the whole protein surface. Based on the obtained results, three preferential regions were selected for further analysis. Accordingly, the docking spheres were centered at the residue: Tyr157, Lys190, and Lys195. The Tyr157 region was chosen as it is the binding site for the reference ligand (the nitrosyl ruthenium complex in the crystal structure PDB ID 7DL4), although the first stage of the experiment did not indicate the possibility of studied complex binding in this binding pocket.

**Figure 9:**
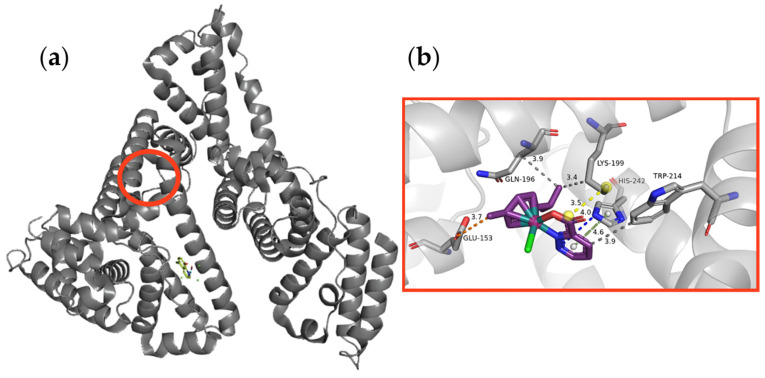
Human serum albumin (HSA) with nitrosyl ruthenium complex (PDB ID 7DL4) and predicted binding pocket (red circle) for the studied ruthenium complex (**a**). Binding modes for the highest-scored pose of complex **1** (**b**) with interactions stabilizing the protein–ligand adduct (key amino acids shown as capped sticks).

According to the scoring function results, the Lys195 region is the most preferred by the investigated ruthenium complex (Figure 9a). The highest-scored pose for complex **1** received a ChemPLP score of 45.88, while the score for the reference ligand was 42.29. Interestingly, the scoring in the Tyr157 region was 45.70 for complex **1** and 52.33 for the nitrosyl ruthenium complex, respectively. For the highest-scoring pose in the preferential binding site, the binding mode was defined.

Complex **1** (Figure 9b) can be stabilized by a salt bridge or N–H⋯O hydrogen bond with Lys199. Moreover, the carbonyl oxygen of the carboxylic group of Glu153 serves as an acceptor in a weak C–H⋯O hydrogen bond, and short contacts between hydrophobic fragments of the ligand and Trp214, Lys199, Gln196, and Ser192 are observed. This ligand forms C–H⋯π contacts with His242. Overall, the obtained data indicate that the adduct of HSA and complex **1** can be formed and stabilized by several intermolecular interactions with the detected protein binding site.

#### 2.9.2. Human Serum Albumin Fluorescence Quenching Assay

The intrinsic fluorescence of tryptophane of the HSA was used for quenching studies, which indicates the affinity between examined compounds and HSA. Fluorescence quenching refers to the process whereby the fluorescence intensity of a fluorophore decreases due to various molecular interactions. This phenomenon encompasses several categories of excited-state reactions, including molecular rearrangements, energy transfer, ground-state complex formation, and collisional quenching. Quenching mechanisms can be classified into two main types: dynamic quenching and static quenching. Dynamic quenching occurs when the fluorophore and the quencher interact during the transient existence of the excited state. In contrast, static quenching is associated with the formation of a fluorophore–quencher complex. Typically, dynamic and static quenching can be differentiated by their respective excited state lifetimes [44]. Given that fluorescence intensity is correlated with quencher concentration, a quenched fluorophore can serve as an effective indicator of the presence of a quenching agent [45]. Consequently, varying concentrations of ruthenium (Ru) complexes were utilized to investigate the quenching properties associated with Ru complexes binding to human serum albumin (HSA). The results indicate that as the concentration of compounds (0.0078–0.5 mM) increases, with a fixed concentration of HSA (10^−7^ M), the intrinsic fluorescence (344 nm) of HSA significantly decreases upon binding with the examined complexes.

**Figure 10:**
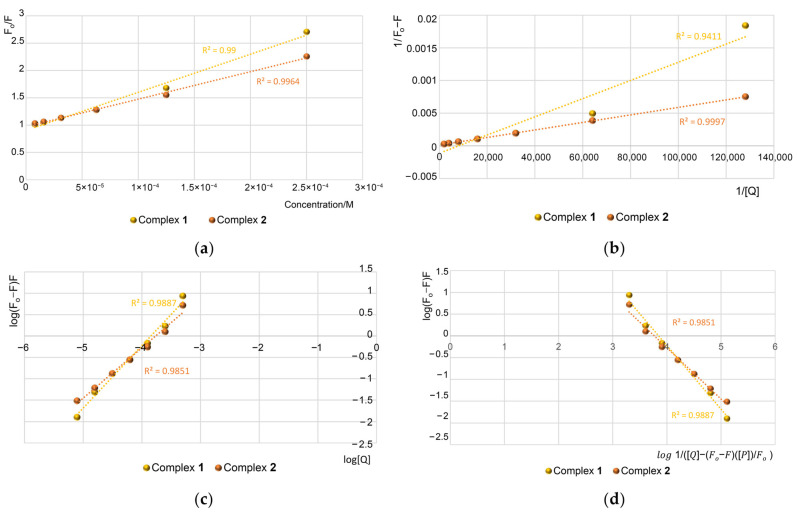
The regression plots for quenching of human serum albumin (HSA) by ruthenium compounds: (**a**) the Stern–Volmer plot (**b**) the Linweaver–Burk plot (**c**) the double logarithm plot, and (**d**) modified double logarithm plot.

Figure 10a displays the Stern–Volmer plots of the quenching of HSA fluorescence by Ru complexes. As it is seen, the plot of F_o_/F for HSA versus [Q] ranging from 7.8 × 10^−6^ M^−1^ to 500 × 10^−6^ M^−1^ is linear. This may suggest that a single quenching mechanism, either static or dynamic is observed at these concentrations [46,47].

The binding constant (K_sv_) was determined to be 6.97 × 10^3^ M^−1^ for compound **1** and 5.03 × 10^3^ M^−1^ for compound **2**. Compared to our previous study [4], these K_sv_ values indicate a reduced capacity for protein binding in the tested compounds. According to Equation (S1) (see ESI), the quenching rate constant (K_q_) values are on the order of 10^11^ M^−1^ s^−1^ for complexes **1** and **2** (Table 5). Reference [48] reports that the maximum scatter collision-quenching constant (K_q_) for various quenchers interacting with biopolymers is 2 × 10^10^ M^−1^ s^−1^. Consequently, the K_q_ values for the protein quenching induced by complexes **1** and **2** exceed this maximum scatter collision-quenching constant. This suggests that the quenching mechanism is not dominated by dynamic collision but rather arises from the formation of a complex. The association constant (K_a_) can be derived from the slope of the Lineweaver–Burk plot of (1/F_o_ − F) against 1/[Q] (Equation (S2), ESI, Figure 10b). The linear relationship observed in these parameters supports the notion of static quenching [49]. Based on these findings, it can be inferred that ruthenium complexes may be stably bound and transported by proteins within biological systems. Further analysis of the fluorescence data allows us to ascertain both the number of binding sites (n) and the binding constants (K_b_). Thus, we used the double logarithm regression curve (Equation (S3), ESI). The corresponding values were obtained from the slope (n) and the intercept (K_b_) of the plot of log [(F_o_ − F)/F] versus log [Q] (Figure 10c). The results showed that the binding constants for **1** and **2** are not similar in order to magnitude. The n values of 1.46 and 1.18 show one-to-one interaction. The modified double logarithm regression curve generated by the plot of log (F_o_ − F)/F vs. log(1/([Q] − (F_o_ − F)[P]/F_o_)) allows us to obtain the binding stoichiometry n and the constant K_b_ (Equation (S4), ESI, Figure 10d). The results showed that the binding constants are smaller than values obtained from Equation (S3) (equation in ESI) (Table 5). In order to evaluate the interaction forces of complex **1** and **2** with HSA, the thermodynamic parameter was also calculated (Equations (S5) and (S6), ESI). The negative value of ΔG^o^ reveals that the interaction process is spontaneous (Table 5).

### 2.10. In Silico Pharmacokinetic and Drug-Likeness Predictions (ADME) for Ruthenium Complexes

In addition, we made an attempt to predict the pharmacokinetic properties of the examined ruthenium complexes (**1** and **2**) using the SwissADME web tool [50]. Bearing in mind the selected properties of these two new compounds with regard to Lipinski’s rule of five (Ro5, Table 6), the complexes meet all conditions with no violation [51]. However, from the point of view of medicinal chemistry, the limit value of molar mass has been exceeded. The tested complexes also do not violate the criteria of Veber’s rule [52,53]. Moreover, the low values gained from the topology of polar surface area, of 88.24 Å^2^ and 44.12 Å^2^, and thus below 140 Å^2^, indicate their good permeability through the membrane (Table 6) [52,53]. The results indicate that Ru compounds could probably be absorbed through the gastrointestinal tract [51]. The Boiled-Egg diagram for the ruthenium complexes is illustrated in Figure S6. Based on this diagram, it is predicted that the Ru compound located in the BOILED-Egg’s white could be passively absorbed in GIT (complex **2**) and that the yolk would be able to passively permeate through the blood–brain barrier (complex **1**).

## 3. Materials and Methods

Information regarding the materials, the synthesis of complexes **1**, **2**, and **3**, as well as the equipment and physicochemical experimental methods, can be found in the Supporting Information. References for materials and methods [3,4,44,54,55,56,57,58,59,60,61,62,63,64,65,66,67,68,69,70,71,72,73] are cited in the Supplementary Materials. A summary of the key structural parameters of the synthesized compounds is presented in Table S3 (Electronic Supplementary Materials). Additional crystallographic data for complexes **1**, **2**, and **3** can be accessed at no charge from The Cambridge Crystallographic Data Centre, with CCDC reference numbers 2339309, 2339304, and 2339306, respectively, via the website www.ccdc.cam.ac.uk/data_request/cif (accessed on 28 October 2024). Details on the biological testing procedures are also included in the Supplementary Information.

## 4. Conclusions

In summary, we have synthesized three ruthenium complexes in different oxidation states (**1**–**3**) with pyridazine-3-carboxylic acid. The compounds were characterized by spectroscopy, and their crystal structures were determined by single-crystal X-ray diffraction analysis. Based on the obtained data, we established that pdz-3-COOH coordinates to the Ru(II) ion in an N,O-bidentate manner. In the case of complexes **2** and **3**, pdz-3-COOH acts as a bridging tridentate ligand connecting two centers—ruthenium and sodium—resulting in a polymeric structure. The hydrogen bonds present in the crystal structures of complexes (especially O–H⋯O, C–H⋯O, and C–H⋯Cl), as well as π-π stacking interactions, contribute to the stabilization of the crystal packing. The results of cyclic voltammetry and magnetic measurements confirmed the +II oxidation state of Ru in complex **1**, and +III in complexes **2** and **3**. Compound **1** exhibits reducing properties, while compounds **2** and **3** have oxidizing properties. These valuable properties can be crucial for explaining the mechanism of action and biological target identification. The comparison of the biological activity of the complexes towards planktonic and biofilm forms of bacteria demonstrates that the previously observed trends continue. The obtained results illustrated that both communities responded contrastingly. Specifically, the response of biofilm *P. aeruginosa* PAO1 to treatment with Ru complexes was significantly stronger. The compounds are able to inhibit biofilm formation by an average of 65% at concentrations ranging from 0.03125 to 0.5 mM. It is worth emphasizing that the tested compounds significantly contribute to reducing pyoverdine secretion, which consequently leads to a reduction in the virulence of the *P. aeruginosa* PAO1 strain. However, surprisingly, the compromising pyoverdine production is not sufficient to inhibit biofilm mass formation significantly (at the level, we expected). Further research is required to explain this process. In this work, the interactions between Ru complexes and their potential transporter, human serum albumin, have also been evaluated. The study was performed by assessing the fluorescence quenching caused by the formation of the drug–protein complex. This technique is well suited to selectively evaluate the interactions observed near the protein fluorophores, particularly Trp214 in HSA. The use of the SV, DLSV, and Hill equations allowed us to determine the binding parameters (on the order of 10^4^ M^−1^) that show similarities with slight differences. Molecular docking supported the results that hydrophobic forces and electrostatic interactions play a substantial role in the binding of complex **1** to HSA. The binding process is governed by weak van der Waals forces, hydrogen bonding, and hydrophobic interactions. The integration of information derived from the synergic combination of different experimental and computational techniques is valuable because the image of HSA binding studies (binding site, binding forces, and type of binding) becomes complete. In addition, computational simulations revealed that new Ru complexes are characterized by promising bioavailability scores and good membrane permeability, making them potential anti-biofilm agents.

## Figures and Tables

**Table 1:** Selected bond lengths (Å) and angles (°) for complex **1**.

Bond Lengths (Å)			
Ru1–N2	2.053(5)	Ru1–Cl1	2.4126(2)
Ru1–O8	2.083(4)	Ru1–Ct1	1.6567(5)
**Valence Angles (°)**			
N2–Ru1–O8	78.10(2)	N2–Ru1–Ct1	133.09(1)
N2–Ru1–Cl1	83.08(2)	O8–Ru1–Ct1	129.80(1)
O8–Ru1–Cl1	86.30(1)	Cl1–Ru1–Ct1	128.42(4)

Ct1 denotes centroid of C10–C11–C12–C13–C14–C15 ring (*p*-cymene).

**Table 2:** Selected bond lengths (Å) and angles (°) for complex **2**.

Bond Lengths (Å)			
Ru1–N2	2.0304(9)	Na1–O2	2.3827(1)
Ru1–O8	2.0327(8)	Na1–O8	2.4897(8)
Ru1–Cl1	2.3352(3)	Na1–N1_(−x+1/2,−y+1/2,−z+1)_	2.5385(1)
		Na1–Na1_(−x+1,−y+1/2,z)_	3.6636(2)
**Valence Angles (°)**			
N2_(−x+1,−y+1/2,z)_–Ru1–N2	180.0		
N2–Ru1–O8_(−x+1,−y+1/2,z)_	99.97(3)	O2–Na1–O2_(−x+1,−y+1/2,z)_	79.51(5)
N2–Ru1–O8	80.03(3)	O2–Na1–O8	80.29(2)
O8_(−x+1,−y+1/2,z)_–Ru1–O8	180.0	O2–Na1–O8_(x,−y+1/2,−z+3/2)_	114.31(3)
N2–Ru1–Cl1	92.92(3)	O8–Na1–O8_(x,−y+1/2,−z+3/2)_	161.81(5)
O8–Ru1–Cl1	90.15(3)	O2–Na1–N1_(−x+1/2,−y+1/2,−z+1)_	158.86(2)
N2–Ru1–Cl1_(−x+1,−y+1/2,z)_	87.08(3)	O8–Na1–N1_(−x+1/2,−y+1/2,−z+1)_	80.18(3)
O8–Ru1–Cl1_(−x+1,−y+1/2,z)_	89.85(3)	O2–Na1–N1_(−x+1/2,y,z+1/2)_	101.38(3)
Cl1–Ru1–Cl1_(−x+3/2,−y+1/2,−z+3/2)_	180.0	O8–Na1–N1_(−x+1/2,y,z+1/2)_	86.46(3)
	N1_(−x+1/2,−y+1/2,−z+1)_–Na1–N1_(−x+1/2,y,z+1/2)_		85.39(5)

**Table 3:** Selected bond lengths (Å) and angles (°) for complex **3**.

Bond Lengths (Å)			
Ru1–N2	2.0311(1)	Na1–O2	2.2996(2)
Ru1–O8	2.0324(1)	Na1–O1	2.3507(2)
Ru1–Cl1	2.3402(4)	Na1–O18	2.3833(1)
Ru2–N12	2.0316(1)	Na1–O8	2.4498(1)
Ru2–O18	2.0388(1)	Na1–N1_(−x+3/2,−y+1/2,−z+3/2)_	2.5726(2)
Ru2–Cl2	2.3285(5)	Na1–N11	2.6265(2)
**Valence Angles (°)**			
N2_(−x+3/2,−y+1/2,−z+3/2)_–Ru1–N2	180.00(9)	Cl2_(−x+3/2,−y+1/2,−z+3/2)_–Ru2–Cl2	180.0
N2–Ru1–O8_(−x+3/2,−y+1/2,−z+3/2)_	100.00(5)	O2–Na1–O1	88.52(6)
N2–Ru1–O8	80.00(5)	O2–Na1–O18	101.28(6)
O8_(−x+3/2,−y+1/2,−z+3/2)_–Ru1–O8	180.00(5)	O1–Na1–O18	108.92(5)
N2–Ru1–Cl1	89.26(4)	O2–Na1–O8	89.81(6)
O8–Ru1–Cl1	92.55(3)	O1–Na1–O8	78.77(5)
N2–Ru1–Cl1_(−x+3/2,−y+1/2,−z+3/2)_	90.74(4)	O18–Na1–O8	166.48(5)
O8–Ru1–Cl1_(−x+3/2,−y+1/2,−z+3/2)_	87.45(3)	O2–Na1–N1_(−x+3/2,−y+1/2,−z+3/2)_	168.31(6)
Cl1–Ru1–Cl1_(−x+3/2,−y+1/2,−z+3/2)_	180.0	O1–Na1–N1_(−x+3/2,−y+1/2,−z+3/2)_	83.39(5)
N12–Ru2–N12_(−x+3/2,−y+1/2,−z+3/2)_	180.00(6)	O18–Na1–N1_(−x+3/2,−y+1/2,−z+3/2)_	89.35(5)
N12–Ru2–O18_(−x+3/2,−y+1/2,−z+3/2)_	79.83(5)	O8–Na1–N1_(−x+3/2,−y+1/2,−z+3/2)_	80.37(4)
N12–Ru2–O18	100.17(5)	O2–Na1–N11	92.19(6)
O18_(−x+3/2,−y+1/2,−z+3/2)_–Ru2–O18	180.0	O1–Na1–N11	170.16(5)
N12–Ru2–Cl2_(−x+3/2,−y+1/2,−z+3/2)_	90.63(4)	O18–Na1–N11	80.57(5)
O18–Ru2–Cl2_(−x+3/2,−y+1/2,−z+3/2)_	90.95(4)	O8–Na1–N11	91.41(5)
N12–Ru2–Cl2	89.37(4)	N1_(−x+3/2,−y+1/2,−z+3/2)_–Na1–N11	94.36(5)
O18–Ru2–Cl2	89.05(4)		

**Table 4:** Electrochemical data [in V vs. Ag/AgCl] of the Ru complexes obtained by CV.

Complex	Scan Rate mV/s	Ru(IV) ↔ Ru(III)				Ru(III) ↔ Ru(II)				Ru(II) ↔ Ru(I)			
		*E*_pa_	*E*_pc_	Δ*E*_p_	*E*_1/2_	*E*_pa_	*E*_pc_	Δ*E*_p_	*E*_1/2_	*E*_pa_	*E*_pc_	Δ*E*_p_	*E*_1/2_
**1**	50					0.780	0.720	0.060	0.750				
	100					0.780	0.720	0.060	0.750				
	200					0.790	0.710	0.080	0.750				
	500					0.795	0.705	0.090	0.750				
**2**	50	1.435	1.345	0.090	1.390	0.225	0.160	0.065	0.193	−0.240	−0.300	0.060	−0.270
	100	1.440	1.350	0.090	1.395	0.230	0.160	0.070	0.195	−0.245	−0.310	0.065	−0.278
	200	1.450	1.360	0.090	1.405	0.240	0.165	0.075	0.203	−0.245	−0.315	0.070	−0.280
	500	1.465	1.375	0.090	1.420	0.245	0.170	0.075	0.208	−0.250	−0.320	0.070	−0.285
**3**	50					0.165	0.115	0.050	0.140	−0.300	−0.425	0.125	−0.363
	100					0.170	0.110	0.060	0.140	−0.290	−0.430	0.140	−0.360
	200					0.180	0.105	0.075	0.143	−0.285	−0.440	0.155	−0.363
	500					0.190	0.100	0.090	0.145	−0.280	−0.450	0.170	−0.365

Δ*E*_p_ = |*E*_pa_ − *E*_pc_|, *E*_1/2_ = ½(*E*_pc_ + *E*_pa_).

**Table 5:** The Stern–Volmer constants(K_sv_), quenching rate constants (K_q_), binding constants (K_b_), number of binding sites (n), and thermodynamic parameter (ΔG) related to the interaction of HSA with the studied Ru compounds.

Compound	Quenching		Binding			Thermodynamic
	K_SV_ [M^−1^]	K_q_ [M^−1^ s^−1^]	K_b_ * [M^−1^]	K_b_ ** [M^−1^]	n	ΔG [kJ mol^−1^]
**1**	6.97 × 10^3^	5.4 × 10^11^	4.23 × 10^5^	7.03 × 10^3^	1.46	−31.45
**2**	5.03 × 10^3^	4.4 × 10^11^	2.78 × 10^4^	5.88 × 10^3^	1.18	−24.84

* according Equation (S3); ** according Equation (S4); (equations in ESI).

**Table 6:** Predicted physicochemical properties, pharmacokinetics, and drug-likeness features of investigated complexes **1** and **2** by the SwissADME server [50].

	Descriptor	Compound	
		1	2
Pharmacokinetics	GI absorption	High	High
	BBB permeability	Yes	No
	P-gp substrate	Yes	Yes
	Water solubility (ESOL)	moderately soluble	moderately soluble
Drug-Likeness	Lipiński	Yes, 0 violation	Yes, 0 violation
	Veber	Yes	Yes
	Bioavability score	0.55	0.55
	TPSA [Å^2^]	44.12	88.24
Physicochemical Properties (Ro5)	Number of H-Bond Acceptors	3	6
	Number of H-Bond Donors	0	0
	Log P_o/w_ (MLOGP)	2.61	0.15
	MW [g/mol]	389.80	418.16

## Data Availability

Data are contained within the article and Supplementary Materials.

## Supplementary Materials

The following supporting information can be downloaded at: https://www.mdpi.com/article/10.3390/molecules29235694/s1, Figure S1: IR spectra of pyridazine-3-carboxylic acid and ruthenium complexes **1**–**3**; Figure S2: UV-Vis spectra of pyridazine-3-carboxylic acid and ruthenium complexes **1**–**3** in distilled water at 298 K; Figure S3: UV-Vis spectra of complexes **1** (a), **2** (b) and **3** (c) in an aqueous solution, after preparation and after 24 h; Figure S4: The plot showing the comparison of the percentage of common intermolecular contacts, occurring in all the tested ruthenium complexes; Figure S5: Metabolic activity of CHO-K1 (A) and A549 (B) cells treated with the ruthenium complexes for 24 h, determined using the MTS test; Figure S6: The Bolied–Egg diagram for the examined Ru complexes (generated by the SwissADME server accessed on 8 October 2024); Table S1: The characteristic IR absorption frequencies (cm^−1^) of the ligand and the ruthenium complexes; Table S2: UV-Vis spectroscopic data for the ligand and the ruthenium complexes; Table S3: Crystallographic data and structure refinement details for the ruthenium complexes in different oxidation states; Table S4: Geometry of classical hydrogen bonds and supramolecular interactions of C–H⋯Cl, as well as Y–X⋯π type interaction for complex **1**, **2** and **3** (Å, °); Table S5: Results of the minimum inhibitory concentration (MIC) for the compounds evaluated, expressed in mM and μg/mL.
