# Supplementary material for: Ruthenium Complexes with Pyridazine Carboxylic Acid: Synthesis, Characterization, and Anti-Biofilm Activity
Source: Molecules. 2024 Dec 2;29(23):5694. doi: 10.3390/molecules29235694 (PMC11643884; doi:10.3390/molecules29235694)
Supplement: Supplementary file 1 [file molecules-29-05694-s001.zip › molecules-3314902-supplementary.pdf]

Electronic Supplementary Information

Ruthenium Complexes with Pyridazine Carboxylic Acid: Synthesis, Characterization, and Anti-biofilm Activity

Patrycja Rogala[*1], Agnieszka Jabłońska-Wawrzycka[*1], Grzegorz Czerwonka[2], Maciej Hodorowicz[3], Sławomir Michałkiewicz[1], Justyna Kalinowska-Tłuścik[3], Marta Karpiel[3,4], Katarzyna Gałczyńska[2]

[1] *Institute of Chemistry, Jan Kochanowski University, 7 Uniwersytecka Str., 25-406 Kielce, Poland*
[2] *Institute of Biology, Jan Kochanowski University, 7 Uniwersytecka Str., 25-406 Kielce, Poland*
[3] *Faculty of Chemistry, Jagiellonian University, 2 Gronostajowa Str., 30-387 Cracow, Poland*
[4] *Doctoral School of Exact and Natural Sciences, Jagiellonian University, 11 Lojasiewicza Str., 30-348 Cracow, Poland.*
Correspondence: patrycja.rogala@ujk.edu.pl; agnieszka.jablonska-wawrzycka@ujk.edu.pl

CONTENTS

1. Experimental section..S2 – S7

2. Supporting tables..S8 – S11

3. Supporting figures...S12 – S16

4. Supporting references..S17 – S18

1. Experimental section

1.1. Materials

$RuCl_3 \cdot xH_2O$, (*p*-cym)ruthenium(II) chloride dimer and pyridazine-3-carboxylic acid were purchased from Sigma Aldrich and used as received. Analytical-grade solvents (methanol, ethanol, hydrochloric acid) were sourced from commercial vendors and used without further purification. The starting (mother) ruthenium(III) chloride solution (0.1 M) was prepared according to the procedure described in the literature [54].

1.2. Syntheses of ruthenium complexes

1.2.1. Preparation of $[(\eta^6\text{-}p\text{-cym})Ru^{II}Cl(pdz\text{-}3\text{-}COO)]$ (1)

The complex **1** was synthesized by mixing the pyridazine-3-carboxylic acid and $[(\eta^6$-*p*-cymene)Ru(μ-Cl)Cl$]_2$ in 2:1 mole ratio, respectively. The ligand (0.4 mmol; 0.0501 g) was dissolved in 20 cm^3 of methanol while ruthenium(II) precursor (0.2 mmol; 0.1247 g) was dissolved in 10 cm^3 of methanol. Then, the two solutions were mixed and refluxed for 10 hours at 65°C. The resulting mixture was allowed to evaporate slowly at room temperature and diffraction quality single crystals were obtained after one week. The crystals were filtered and dried in air. The compound was collected in 78% yield. (Found: C, 45.47; H, 4.40; N, 6.91; Calc. for $RuClC_{15}H_{17}N_2O_2$: C, 45.75; H, 4.35; N, 7.11%). IR: 3082(m), 3066(m), 2968(ms), 2926(m), 1657(vs), 1581(ms), 1552(ms), 1505(ms), 1470(ms), 1449(s), 1380(s), 1333(vs), 1221(s), 1158(ms), 1146(s), 1111(ms), 1079(s), 1054(ms), 1041(ms), 876(s), 852(s), 781(vs), 716(ms), 677(ms), 657(ms), 540(ms), 448(s). ^{1}H NMR (400 MHz, DMSO-d_6, δ ppm): 1.12 (6H, *d*, –CH(C**H**$_3$)$_2$, J = 6.9 Hz); 2.19 (3H, *s*, C**H**$_3$); 2.73 (1H, *m*, –C**H**(CH$_3$)$_2$, J = 6.9 Hz); 5.83–6.11 (4H, *d*, C**H** (p-cymene), J = 6.4 Hz); 7.93 (1H, *d*, **H5**-L, J = 7.4 Hz), 8.51 (1H, *d*, **H4**-L, J = 7.5 Hz), 9.54 (1H, *d*, **H6**-L, J = 5.4 Hz), ^{13}C NMR (100 MHz, DMSO-d_6, δ ppm): 18.29 (**C**H$_3$); 21.85 (–CH(**C**H$_3$)$_2$); 30.69 (–**C**H(CH$_3$)$_2$); 79.36–101.07 (**C**H (p-cymene)); 124.86 (**C4**-L); 129.43(**C5**-L); 152.28 (**C6**-L); 154.57 (**C3**-L); 170.24 (**C7**-L). μ_{eff} = 0 μ_B for a low-spin Ru^{2+} (electron configuration of $_{44}Ru^{2+}$ [Kr] $4d^6$).

1.2.2. Preparation of $[Ru^{III}Cl_2(pdz\text{-}3\text{-}COO)_2Na(H_2O)]_n(H_2O)_{0.11}$ (2) and $[Ru^{III}Cl_2(pdz\text{-}3\text{-}COO)_2Na(H_2O)_2]_n$ (3)

To obtain complexes **2** and **3**, an aqueous solution of $NaHCO_3$ (0.4 mmol, 0.0347 g) was added dropwise to a methanolic solution of pyridazine-3-carboxylic acid (0.4 mmol, 0.0505 g), and the resulting mixture was stirred for 20 min at room temperature. Then, this solution was added to a 0.1 M solution of ruthenium(III) chloride (2 mL, 0.2 mmol). The mixture was heated under reflux at about 65°C with continuous stirring for 10 hours. The red block-shaped crystals (complex **2**) and reddish crystals with a bladed termination (complex **3**) were formed after a few days, which were filtered off and dried in air. The crystals were collected in a yield of 25% and 4%, respectively.

Complex **2** – Found: C, 26.03; H, 1.77; N, 11.75; Calc. for $RuCl_2C_{10}H_8N_4O_5Na$: C, 26.16; H, 1.76; N, 12.02%). IR: 3420(br,s), 3085(m), 1655(vs), 1587(m), 1563(ms), 1457(ms), 1394(s), 1323(vs), 1220(s), 1153(s), 1088(ms), 866(s), 782(s), 726(ms), 667(ms), 573(m), 482(ms), 466(ms).

Complex **3** – IR: 3521(br,s), 3448(br,s), 3073(m), 1675(vs), 1636(vs), 1591(s), 1560(s), 1455(ms), 1390(s), 1312(vs), 1304(vs), 1216(s), 1180(ms), 1152(ms), 1085(m), 1038(m), 860(s), 786(s), 735(ms), 722(ms), 668(ms), 574(m), 482(s), 464(s).

1.3. Physical measurements

The percentage compositions of the elements (C, H and N) of the synthesized complexes were determined using a Vario Micro Cube Elemental Analyser CHNS. The IR spectra were recorded on a Nicolet 380 FT-IR type spectrophotometer in the spectral range 4000–400 cm^{-1} using the KBr discs method. The NMR spectra were recorded on Bruker Avance III instrument equipped with a 5-mm, normal configuration probe with z-axis gradient capability at a field strength of 9.4 T operating at 400.26 and 100.64 MHz for ^{1}H and ^{13}C, respectively, in d_6-DMSO at 25°C. Chemical shifts (δ) are reported relative to internal TMS (^{1}H and ^{13}C) standard. UV-Vis measurements in aqueous solutions were performed on a V-630 UV-Vis spectrophotometer from Jasco using 1 cm cuvettes. All absorbance measurements were recorded at room temperature and the concentrations were: 5.02×10^{-5} M (complex **1**), 3.47×10^{-5} M (complex **2**), 4.30×10^{-5} M (complex **3**), and 1.08×10^{-4} M (ligand). Magnetic measurements were carried out on a magnetic susceptibility balance (Sherwood Scientific) at room temperature by Gouy's method, using $Hg[Co(NCS)_4]$ as a calibrant. The voltammetric experiments were conducted on a Model M161E electrochemical analyser cooperating with the EALab 2.1 software (mtm-anko, Cracow, Poland). In a conventional three-electrode cell, a glassy carbon electrode (GCE) of 2 mm diameter, A = 0.0314 cm^2 (Mineral, Warsaw, Poland) and a platinum wire were used as a working electrode and a counter (auxiliary) electrode, respectively. The potential was measured against the external silver/silver chloride reference electrode (Ag/AgCl) with 1 M NaCl solution (Mineral, Warsaw, Poland). To avoid water leakage, the reference electrode was isolated from the solution by a salt bridge with a frit of Victor Glass. Pure argon was used for deoxygenating the solutions prior to voltammetric investigations. The electrochemical studies of the ruthenium complexes and free ligand were carried out in a mixture of CH_3CN/EtOH (3 : 2, *v/v*; Chempur, analytical-grade) with 0.1 M tetrabutylammonium hexafluorophosphate ($TBAPF_6$) (Fluka, electrochemical grade) as a supporting electrolyte. The concentration of the ligand and the Ru complexes for the cyclic voltammetry measurements was $1\cdot10^{-3}$ M. The measurements were performed at room temperature (25 ± 1°C). Some experiments were performed with the use of differential pulse voltammetry (DPV) on carbon fiber (CF) disk microelectrode (33 μm in diameter (BASi, United Kingdom)). DPV voltammogram was registered using a pulse amplitude of 20 mV, pulse width of 80 ms and scan rate of 20 mV s^{-1}. This technique is considered a convenient method because of its good sensitivity selectivity and resolution of the signals, limited influence of adsorption phenomena on recorded curves and thus excellent reproducibility [55].

1.4. Crystallographic data collection and structure refinement

Diffraction intensity data for the single crystals of the three new compounds were collected at temperatures of 140 K for **1** and 100 K for **2**, **3** on a Rigaku XtaLAB Synergy-S diffractometer with a Mo Kα monochromatic mirror (λ = 0.71073 Å). Cell refinement and data reduction were performed using firmware [56]. The positions of all non-hydrogen atoms were determined using SHELXT software [57]. All non-hydrogen atoms were anisotropically refined using a weighted full least squares matrix on F^2 [58]. All hydrogen atoms attached to carbon atoms were placed in idealized geometries and refined using a driving model with U_{iso}(H) fixed at 1.2 U_{eq} (C_{arom}) and 1.5

U_{eq} (C_{methyl}). In structure **3**, the hydrogen atoms at the water molecules were determined based on a differential Fourier map by additionally introducing DFIX (0.85) and DANG (1.38) restraints on the oxygen O1 and hydrogen atoms in the water molecule. Structure of the compound **1** was refined as an inversion twin with a twin ratio of 0.22:0.78. The crystallographic data and some details of the structural refinement are summarized in Table S1 (ESI). The drawings were made with Diamond ver. 4.6.8 [59]. CCDC software 2339309, 2339304, 2339306, contain additional crystallographic data for **1**, **2** and **3** respectively, and these data can be obtained free of charge from The Cambridge Crystallographic Data Centre via www.ccdc.cam.ac.uk/data_request/cif.

1.5. Hirshfeld surface analysis

Molecular Hirshfeld surfaces calculations were performed using the Crystal Explorer package *ver.* 21.5 [60]. When the .cif file of the title compounds was entered into the Crystal Explorer program, all of the bond lengths to hydrogen were automatically modified to the standard neutron values (CH = 1.083 A). Hirshfeld surface analysis included the descriptor d_{norm} and the *shape index* [61,62]. The calculations and details of analysis were made as described in paper [3]. The molecular Hirshfeld surface of complexes **1**, **2** and **3** were generated using a standard (high) surface resolution with the 3D d_{norm} surfaces mapped over a fixed colour scale of −0.0667 (for **1**)/−1.2729 (for **2**)/−0.0667 (for **3**) (red) to 1.656 (for **1**)/1.1272 (for **2**)/0.0667 (for **3**) Å (blue). The *shape index* was mapped in the colour range of −1 to 1 (for **1**, **2** and **3**). The colour encodes normalized distance to nearest nuclei and thus conveniently illustrates the "strength" of all types of the intermolecular contacts present. In turn, the fingerprint plots provide a quantitative measure of the intermolecular interactions on the surface.

1.6. Microbiological studies

1.6.1. Bacterial strains and cultivation

The *Pseudomonas aeruginosa* PAO1 strain was derived from the International *Pseudomonas aeruginosa* Reference Panel, deposited in the Belgian Co-ordinated Collection of Microorganisms (BCCM)/LMG Bacteria Collection, Ghent University, Gent, Belgium (http://bccm.belspo.be/about-us/bccm-lmg) [63]. Additionally, *Staphylococcus aureus* ATCC 6538P, and *Escherichia coli* ATCC 8739 strains were used as a representative strains for Gram-positive and Gram-negative bacteria, respectively. Bacteria were cultivated overnight (at 37°C with shaking 160 r.p.m. in Ecotron incubator, Infors HT, Basel, Switzerland) in TSB medium (Trypticase Soy Broth, Biocorp, Warsaw, Poland), next diluted with fresh medium in ratio 1:100 (bacteria cell densities ~10^7 CFU/mL) and supplemented with tested compounds. Bacteria were exposed to the following tests.

1.6.2. Minimum Inhibitory Concentration

Minimal Inhibitory Concentration (MIC) was determined by the dilution method using 96-well flat-bottomed transparent microtiter plates (Greiner Monroe, NC, USA). An overnight culture of bacteria (*S. aureus*, *E. coli, P. aeruginosa* PAO1) was diluted at a ratio of 1:100 (0.5 McFarland turbidity) with fresh TSB medium and supplemented with an aqueous solutions of the ruthenium complexes or the ligand (at concentrations in the range from 1 mM to 0.015625 mM), and resazurin (0.02 mg/mL) as a growth indicator [64]. After that, the plates were incubated overnight at 37°C with shaking at 160 r.p.m. Two control tubes were maintained for each test batch. These included: negative control (bacterial culture in the medium without growth inhibitory agent) and positive

control (antibiotic control—ciprofloxacin). The MIC was expressed as the lowest concentration of tested compounds at which no microbial growth was observed.

1.6.3. Inhibition of P. aeruginosa PAO1 biofilm formation

An overnight culture of *P. aeruginosa* PAO1 supplemented with the tested complexes was exposed to adherence and biofilm formation assay. Adhesion was estimated by quantifying adherent cells to 96-well transparent flat-bottomed microtiter plates (Greiner, Monroe, NC, USA) by crystal violet staining (0.01%, w/v). The overnight culture was transferred and diluted in ratio 1:100 with fresh TSB medium (180 μl) into 96-well microtiter plates. Serial dilutions of the tested compounds were performed covering the concentration range from 0.015625 mM to 1 mM. Serial dilutions of ciprofloxacin at a concentration of 1 mg/mL were used as a control. The plates were incubated overnight at 37°C. Next, the residual planktonic cells were washed by immersing whole plate in distilled water. The plates were dried out, filled with 200 μl of the crystal violet solution and incubated for 15 minutes at room temperature (RT). The crystal violet was removed and the plates were washed again with distilled water, in triplicate. The crystal violet from adhered cells was extracted by filling the wells by 96% ethanol (incubation 15 min. at RT) and subjected to absorbance measurement (λ = 595 nm) in an Infinite M200PRO microplate reader (Tecan, Männedorf, Switzerland). The measurement results, expressed in absorbance units, were converted to percentages to allow comparison of numerical data obtained in different experiments.

1.6.4. Pyoverdine determination assay

Pyoverdine determination was performed in TSB medium. The measurement of pyoverdine fluorescence was performed at the excitation wavelength of λ = 398 nm and the emission wavelength of λ = 455 nm, as described previously [4]. Experiments were carried out in triplicate in 96-well black flat-bottomed microtiter plates (Greiner Monroe, NC, USA) for 24 h in cell-free culture medium using an Infinite M200PRO (Tecan, Männedorf, Switzerland). The assay was performed with ligand, complex **1**, complex **2** (the tested concentrations of compounds were: 1–0.015625 mM), and ciprofloxacin was used as negative control.

1.7. Cytotoxicity Activity (MTS Assay)

The Chinese Hamster Ovary (CHO-K1) and the adenocarcinoma human alveolar basal epithelial (A549) line cells were cultured at 37°C in a humidified 5% CO_2 atmosphere in plastic dishes in F-12K medium (Sigma Aldrich Chemicals, Rockville, MD, USA) supplemented with 10% fetal bovine serum (Invitrogen, Carlsbad, CA, USA), 2 mM L-glutamine (Sigma Aldrich Chemicals, USA) and antibiotics (100 units/mL penicillin and 100 μg/mL streptomycin (Invitrogen, CA, USA)) at 37°C. Cytotoxic properties of ruthenium complexes **1** and **2** were measured by a MTS Cell Proliferation Assay Kit (Abcam, ab197010) in accordance with the manufacturer's instructions. Cells were seeded into a 96-well plate and incubated with the ruthenium complexes in the concentration of 250, 125, 60, 30, 15, 7 μM for 24 h at 37°C in a humidified atmosphere of 5% CO_2. After incubation, a solution of MTS (3-(4,5-dimethylthiazol-2-yl)-5-(3-carboxymethoxyphenyl)-2-(4-sulfophenyl)-2H-tetrazolium) was added to each well and incubated at 37°C for 4 h. The measurement of the absorbance of the solution related to the number of live cells was conducted on a TECAN Spark Microplate Reader (TECAN, Männedorf, Switzerland) at 490 nm. All samples were tested in three independent experiments. Results were normalized to the control.

1.8. Molecular docking procedures

The crystal structure of HSA (Human Serum Albumin) with nitrosyl ruthenium complex adduct (PDB ID 7DL4) was downloaded from Protein Data Bank (PDB) [65]. The initial geometry of the studied Ru complex was taken from the final .cif file. The preparation of the protein and ligand structures was conducted using Maestro 13.9 [66] and Mercury 2023.3.0 programs [67]. Molecular docking experiment was performed using GOLD 2023.3.0 (Genetic Optimisation for Ligand Docking) [68] in a semi-flexible docking mode. Molecular docking study was conducted in two steps. First, the docking region was expanded to include all atoms of the HSA protein within a radius of 50 Å. This procedure allowed a search for the most prominent binding sites for the investigated ruthenium complex. The native nitrosyl ruthenium complex of the 7DL4 structure was re-docked as a reference ligand with RMSD 2.959 for the best-scored pose. The number of genetic algorithm runs was set to 50 for each docked compound. In the second stage, the three main docking regions identified during the previous experiment were selected. The docking spheres were centered at Tyr157, Lys190, and Lys195, including all atoms within the 15 Å radius. The number of genetic algorithm runs was set to 20 for each compound. Every step was evaluated based on the reference nitrosyl ruthenium complex results. In the docking procedure, the ChemPLP empirical scoring function was applied [69]. The obtained molecular docking results were analyzed and visualized with PyMOL [70] and Protein-Ligand Interaction Profiler (PLIP) server [71].

1.9. Human serum albumin (HSA) fluorescence quenching assay

The interactions between the ruthenium complexes and the HSA protein were determined. Fluorescence quenching assay was performed according to a previously published procedure [4]. The concentration of HSA was set to 10 μM and the quenching of its fluorescence was performed. The solution of BSA or HSA was mixed with 0, 7.8, 15.6, 31.3, 62.5, 125, 250 or 500 μM of the Ru compounds, and the fluorescence intensity was measured at a fixed wavelength at λ= 344 nm, while the excitation wavelength was λ= 280 nm. The measurements were conducted using Infinite M200PRO microplate reader (Tecan, Männedorf, Switzerland).

Fluorescence quenching is described by the Stern–Volmer equation [72]:

$$\frac{F_o}{F} = 1 + K_q\tau_0[Q] = 1 + K_{SV}[Q] \qquad \text{(S1)}$$

where F_o and F represent the fluorescence intensities in the absence and in the presence of quencher, respectively. K_q is the quenching rate constant of biomolecule, K_{sv} is the dynamic quenching constant, τ_0 is the average lifetime of the biomolecule without quencher (τ_o = 6 ns) [44], and [Q] is the concentration of quencher.
The static quenching equation of Lineweaver–Burk can be used [73]:

$$\frac{1}{F_o - F} = \frac{1}{F_o} + \frac{1}{K_a F_o [Q]} \qquad \text{(S2)}$$

where K_a is the binding constant of Ru complexes with HSA.
There are several models to determine the binding parameters. Two models were used to analyze the received data: double logarithm regression curve and modified double logarithm regression

curve. Using the double logarithm regression curve, the binding constant K_b, and the number of binding stoichiometry n was determined using the Hill equation (S3) [72]:

$$log\frac{F_o-F}{F} = logK_b + nlog[Q] \qquad \text{(S3)}$$

where F_o and F are the steady-state fluorescence intensities at the maximum wavelength in the absence and presence of quencher, respectively, [Q] is the quencher concentration.
The second method, modified double logarithm regression curve, in contrast to the previous one, takes into account the total concentration of protein present in the analyzed solution:

$$log\frac{F_o-F}{F} = nlogK_b + nlog\frac{1}{[Q]-(F_o-F)\frac{[P]}{F_o}} \qquad \text{(S4)}$$

where [P] is HSA concentration.
Based on the binding constants the free energy change can be estimate by van’t Hoff equation:

$$logK_b = \frac{-\Delta H}{RT} + \frac{\Delta S}{R} \qquad \text{(S5)}$$

where K_b is the binding constant at the measuring temperatures and R is the gas constant.
The free energy change (ΔG°) can be calculated by following equation:

$$\Delta G^o = \Delta H^o - T\Delta S^o = -RTlnK_b \qquad \text{(S6)}$$

2. Supporting tables

Table S1. The characteristic IR absorption frequencies (cm^{-1}) of the ligand and the ruthenium complexes.

Assignment	pdz-3-COOH	Complex 1	Complex 2	Complex 3
$\nu_{O\text{-}H}$ (H_2O)	–	–	3420	3521, 3448
$\nu_{C\text{-}H\ aromatic}$	3086; 3062; 2991	3082; 3066	3085	3073
$\nu_{C\text{-}H\ aliph.}$	–	2968, 2926	–	–
ν_{COOH}	1733	–	–	–
$\nu_{as.COO^-}$	–	1657	1655	1676, 1636
$\nu_{s.COO^-}$	–	1333	1323	1312, 1304
$\nu_{C=C,\ C=N,\ N=N}$ skeletal ring	1575; 1560; 1458; 1436	1581; 1552; 1470; 1449	1587; 1563; 1457	1591, 1560, 1455

Table S2. UV-Vis spectroscopic data for the ligand and the ruthenium complexes.

Compound	Transition λ, nm (ε, $dm^3/mol\cdot cm$)	
	π→π*/n→π*	MLCT or LMCT/*d*–*d*
pdz-3-COOH	201 (5576), 251 (1268), 301 (239)	
Complex **1**	203 (16126), 235 (5293), 311 (3602)	386 (1238)
Complex **2**	205 (27630), 241 (8930), 282 (3941)	332 (5641), 401 (4312), 427 (4415)
Complex **3**	203 (23173), 222 (8943), 245 (4115) 269 (1526), 283 (2794)	320 (2794), 341 (2678), 374 (1950), 400 (1936), 435 (1900), 474 (168)

Table S3. Crystallographic data and structure refinement details for the ruthenium complexes in different oxidation states.

	**Complex 1**	**Complex 2**	**Complex 3**
Empirical formula	$C_{15}H_{17}ClN_2O_2Ru$	$C_{10}H_8Cl_2N_4NaO_{5.11}Ru$	$C_{10}H_{10}Cl_2N_4NaO_6Ru$
Formula weight (g/mol)	393.82	460.99	477.18
Temperature (K)	140(2)	100(2)	100(2)
Wavelength of MoKα radiation (Å)		0.71073	
Crystal system, space group	monoclinic, $P\ 2_1$/c	orthorhombic, *C* cca	monoclinic, *I* 2/a
Unit cel dimensions			
a (Å)	12.0385(8)	13.2317(2)	13.7867(9)
b (Å)	10.5595(6)	16.7970(3)	14.1387(7)
c (Å)	12.0846(7)	13.9233(3)	16.3497(8)
β (°)	91.453(5)		104.665(5)
Volume ($Å^3$)	1535.71(2)	3094.49(1)	1686.59(4)
Z, density (calculated) (Mg/m^3)	4; 1.703	8; 1.979	8; 2.056
Absorption coefficient (mm^{-1})	1.200	1.416	1.429
F (000)	792	1807	1880
Crystal size (mm)	0.20 × 0.15 × 0.13	0.20 × 0.14 × 0.10	0.30 × 0.20 × 0.15
Theta range for data collection (°)	2.562 – 30.043	3.522 – 33.603	3.328 – 30.816
Index ranges	–13 ≤ h ≤ 14, –14 ≤ k ≤ 14, 0 ≤ l ≤ 16	–19 ≤ h ≤ 19, –26 ≤ k ≤ 26, –20 ≤ l ≤ 20	–18 ≤ h ≤ 18, –19 ≤ k ≤ 17, –21 ≤ l ≤ 21
Reflections collected/independent [I> 2sigma(I)]	3496/3496 [R_{int} = 0.0363]	89529/2934 [R_{int} = 0.0408]	38279/4308 [R_{int} = 0.0826]
Completeness to θ (%)	2θ = 25.242°; 96.0	θ = 25.242°; 98.4	θ = 25.242°; 99.6
Refinement method		Full-matrix least-squares on F^2	
Data/restraints/parameters	3496/0/194	2934/1/115	4308/4/228
Goodness-of-fit on F^2	1.080	1.078	1.106
Final R indices [I>2sigma(I)]	R_1 = 0.0614, wR_2 = 0.1831	R_1 = 0.0206, wR_2 = 0.0543	R_1 = 0.0286, wR_2 = 0.0684
R indices (all data)	R_1 = 0.0675, wR_2 = 0.1876	R_1 = 0.0235, wR_2 = 0.0557	R_1 = 0.0332, wR_2 = 0.0708
Largest diff. peak and hole ($e·Å^{-3}$)	4.045 and –1.947	0.681 and –0.817	0.914 and –1.057

Table S4. Geometry of classical hydrogen bonds and supramolecular interactions of C–H···Cl, as well as Y–X···π type interaction for complex **1**, **2** and **3** (Å, °).

Complex	D–H···A	D–H (Å)	H···A / X···Cg (Å)	D···A/ Y···Cg (Å)	∠D-H···A/ Y–X···Cg (°)
1	C(6)–H(6)···Cl(1)$_{(-x,-y+1,-z+1)}$	0.95	2.75	3.639(7)	156.2
	C(4)–H(4)···O(8)$_{(x,-y+1/2,z-1/2)}$	0.95	2.45	3.263(8)	144.0
2	C(4)–H(4)···O(9)$_{(-x+1,-y+1,-z+1)}$	0.95	2.40	3.1314(1)	133.8
	C(6)–H(6)···Cl(1)$_{(x,-y+1/2,-z+1/2)}$	0.95	2.85	3.6144(1)	138.2
	C(5)–H(5)···O(9)$_{(x,-y+1,z-1/2)}$	0.95	2.50	3.3619(1)	151.4
	O(2)–H(2)···O(9)	0.933(2)	1.860(2)	2.7731(1)	165(2)
3	O(1)–H(1B)···O(19)$_{(-x+1,-y+1,-z+1)}$	0.85	2.07	2.8862(2)	160.4
	O(2)–H(2B)···O(9)	0.85	2.35	3.027(2)	136.6
	C(5)–H(5)···O(19)$_{(x,-y+1/2,z+1/2)}$	0.95	2.51	3.172(2)	126.6
	C(5)–H(5)···O(1)$_{(x,-y+1/2,z+1/2)}$	0.95	2.49	3.240(2)	136.0
	C(4)–H(4)···Cl(1)$_{(x,-y+1/2,z+1/2)}$	0.95	2.98	3.7389(2)	137.8
	C(16)–H(16)···Cl(1)$_{(-x+3/2,-y+1/2,-z+3/2)}$	0.95	2.73	3.5342(2)	142.9
	C(15)–H(15)···O(9)$_{(-x+3/2,y,-z+2)}$	0.95	2.46	3.269(2)	142.6
	C(14)–H(14)···Cl(1)$_{(x+1/2,y+1/2,z+1/2)}$	0.95	2.83	3.4791(2)	126.2
	C(14)–H(14)···Cl(2)$_{(x,-y+3/2,z+1/2)}$	0.95	2.91	3.7704(2)	150.5
	O(2)–H(2A)···Cl(1)$_{(-x+1,y+1/2,-z+3/2)}$	0.83(3)	2.61(3)	3.3931(2)	159(3)
	O(2)–H(2A)···Cl(2)$_{(-x+3/2,-y+3/2,-z+3/2)}$	0.83(3)	2.95(3)	3.4039(2)	117(2)
	C7–O9···Cg2$_{(-1/2+x,1-y,z)}$		3.6337(2)	4.574(2)	134.88(1)

Cg2 denotes centre of gravity of N11–N12–C13–C14–C15–C16 ring (pyridazine)

Table S5. Results of the minimum inhibitory concentration (MIC) for the compounds evaluated, expressed in mM and μg/mL.

**Compound**	**BACTERIA**					
	S. aureus		***E. coli***		***P. aeruginosa* PAO1**	
	mM	μg/mL	mM	μg/mL	mM	μg/mL
$RuCl_3·xH_2O$	> 1	>207	> 1	>207	> 1	>207
Ru(II) precursor	> 1	> 612	> 1	> 612	> 1	> 612
pzd-3-COOH	> 1	> 124	> 1	> 124	> 1	> 124
Complex **1**	1	394	1	394	1	394
Complex **2**	1	461	1	461	1	461
Ciprofloxacin	< 0.2	< 62.5	< 0.2	< 62.5	< 0.2	< 62.5

3. Supporting figures

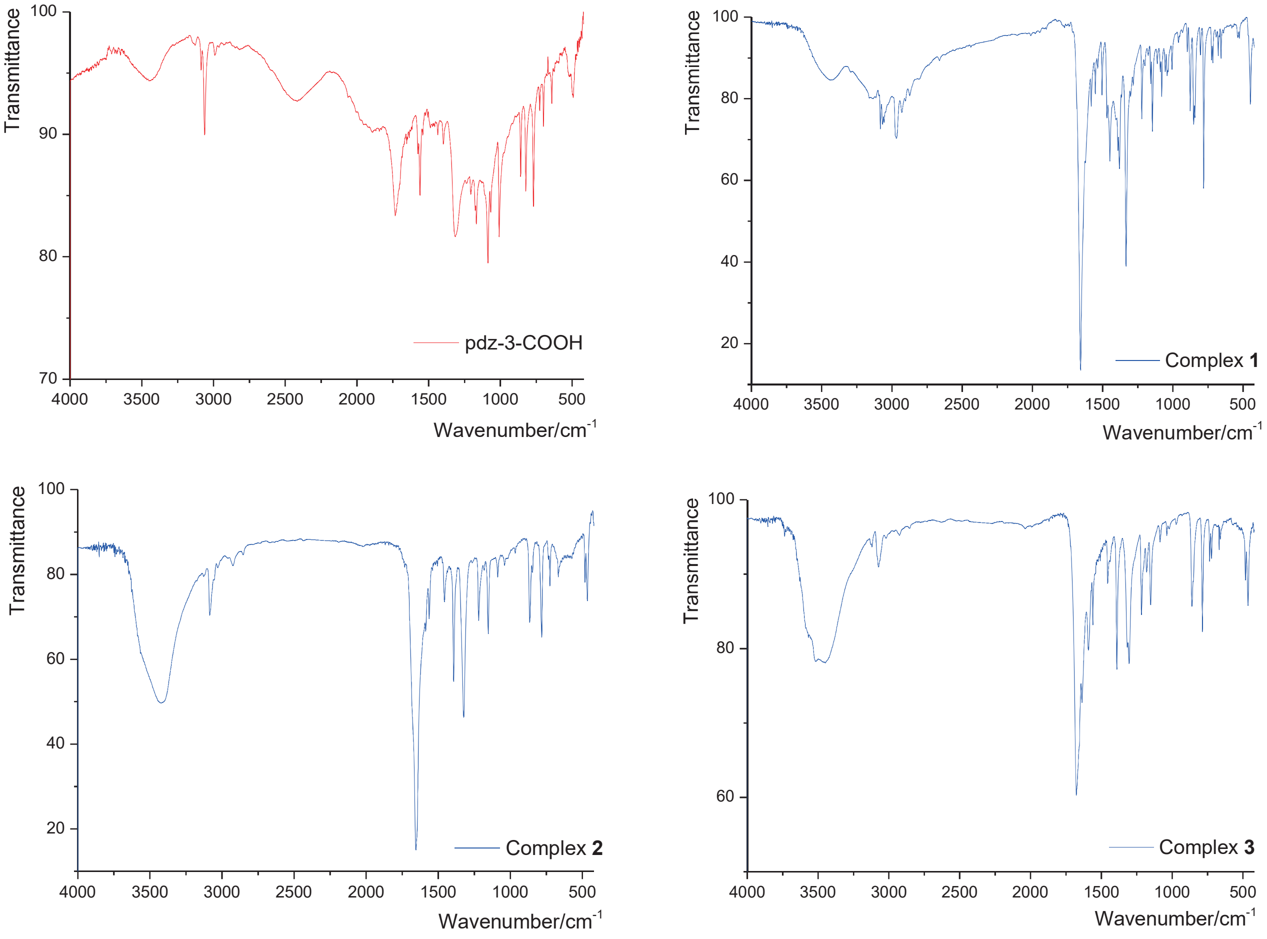

Figure S1. IR spectra of pyridazine-3-carboxylic acid and ruthenium complexes **1–3**.

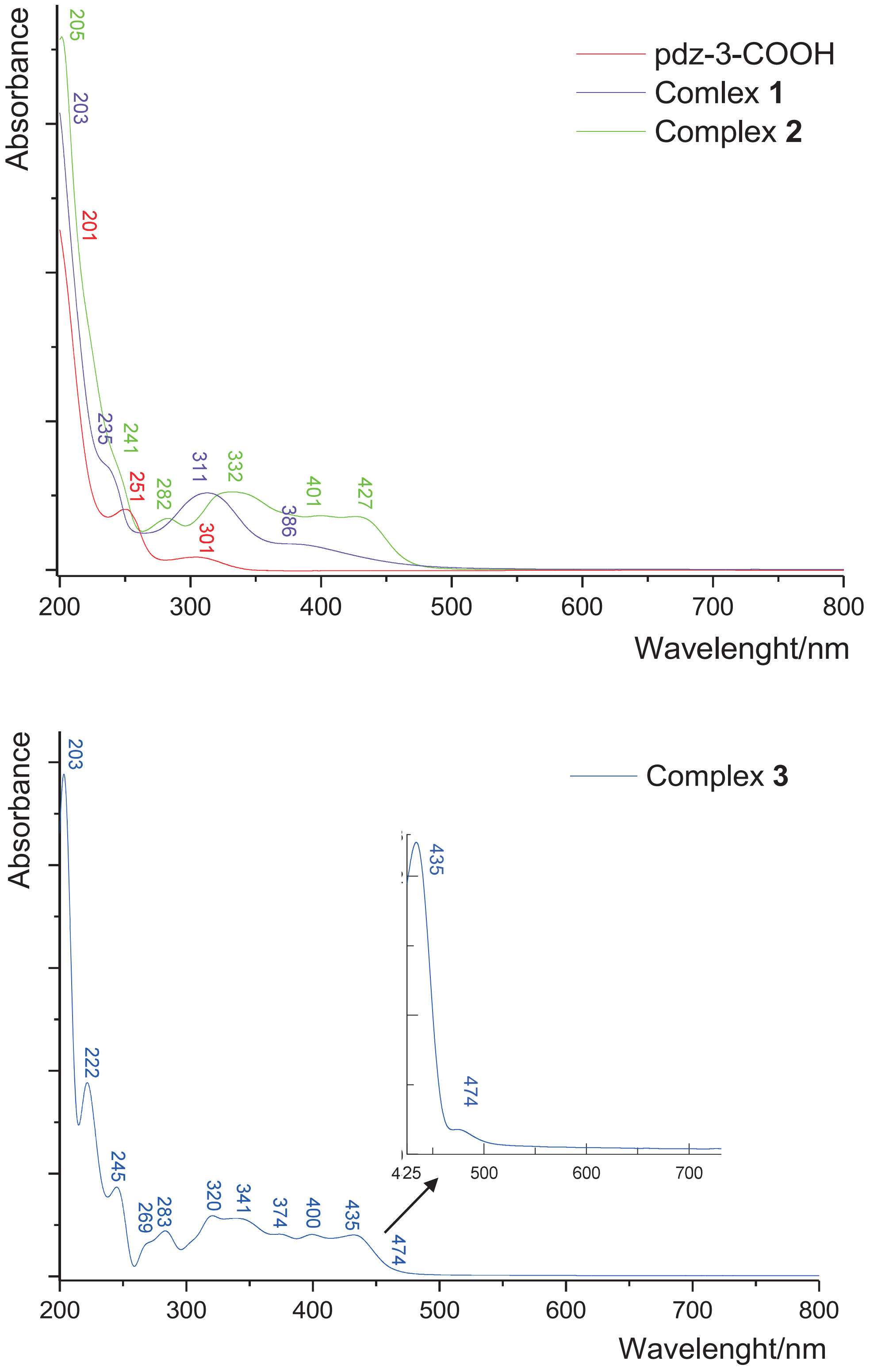

Figure S2. UV-Vis spectra of pyridazine-3-carboxylic acid and ruthenium complexes **1–3** in distilled water at 298 K.

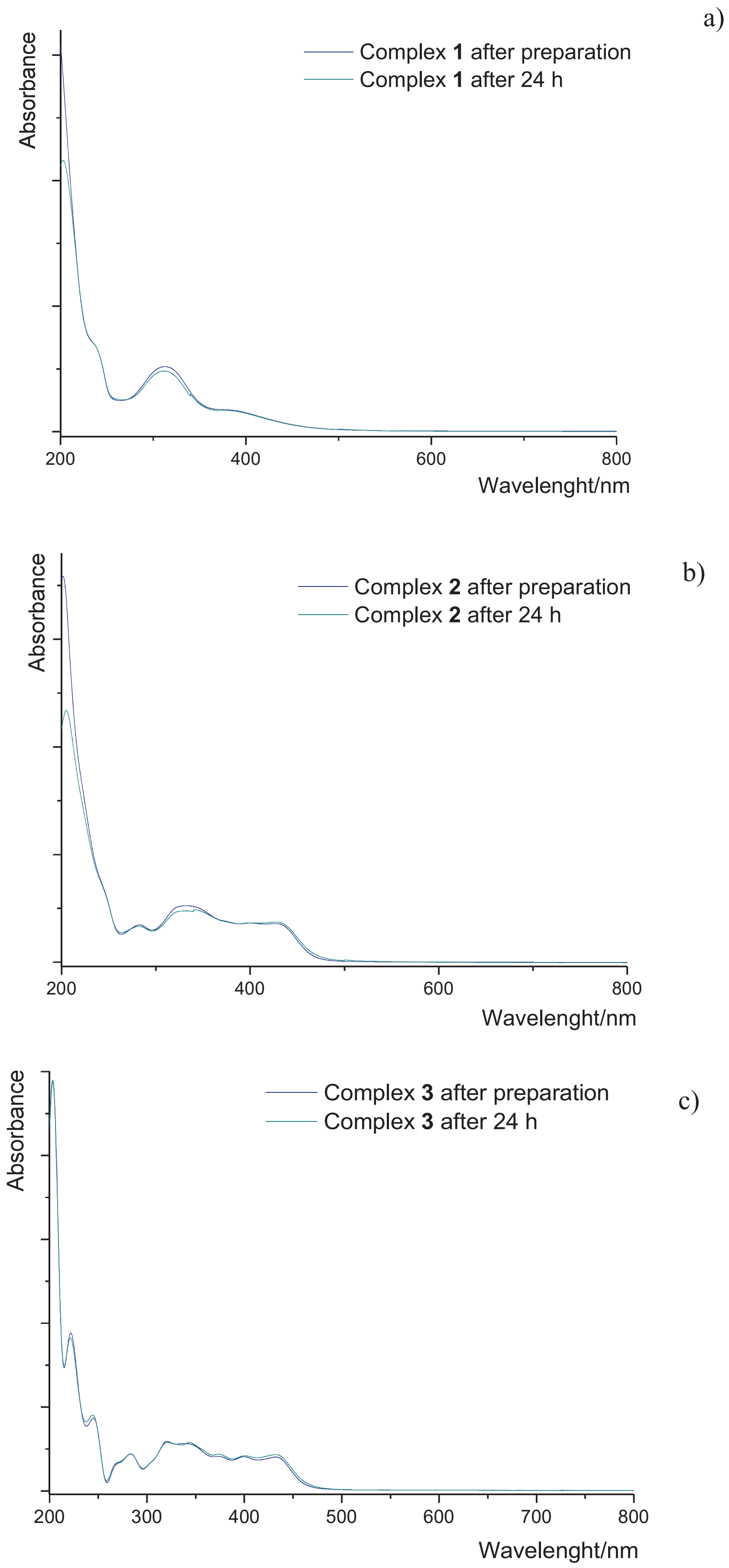

Figure S3. UV-Vis spectra of complexes **1** (a), **2** (b) and **3** (c) in an aqueous solution, after preparation and after 24 h.

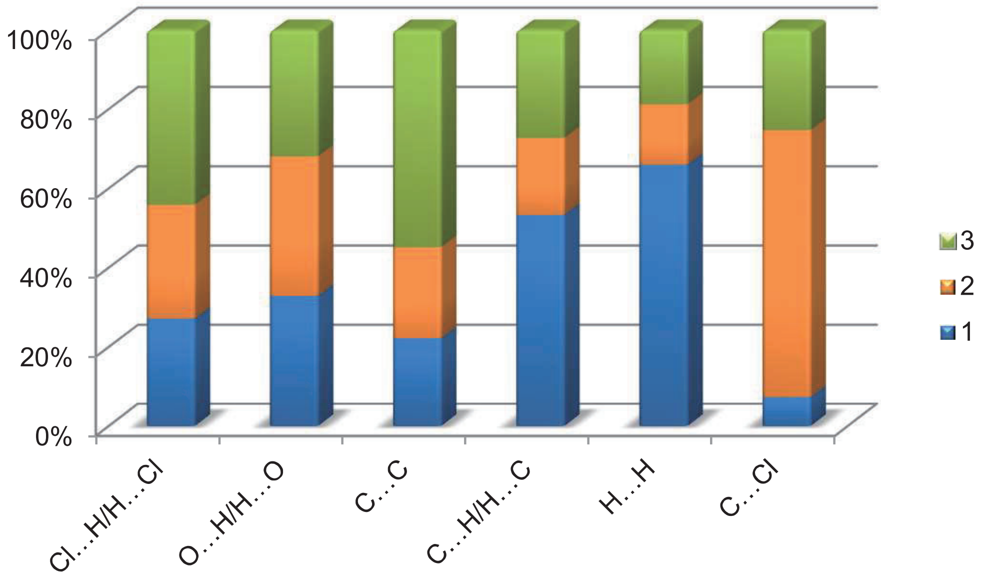

Figure S4. The plot showing the comparison of the percentage of common intermolecular contacts, occurring in all the tested ruthenium complexes.

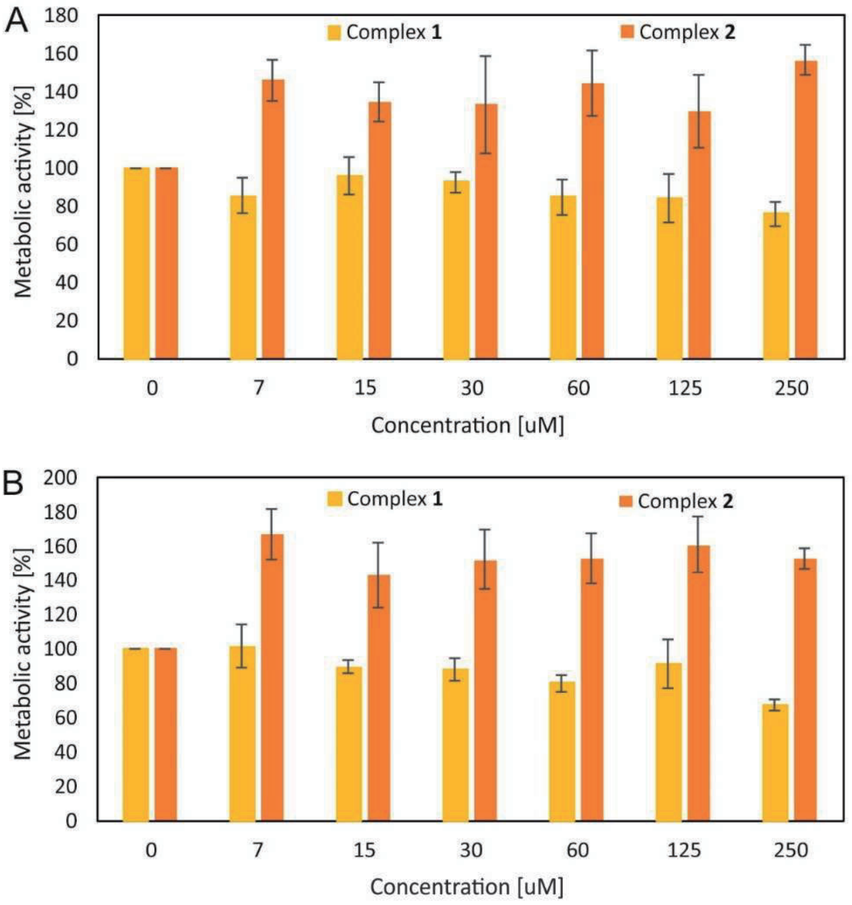

Figure S5. Metabolic activity of CHO-K1 (A) and A549 (B) cells treated with the ruthenium complexes for 24 hours, determined using the MTS test.

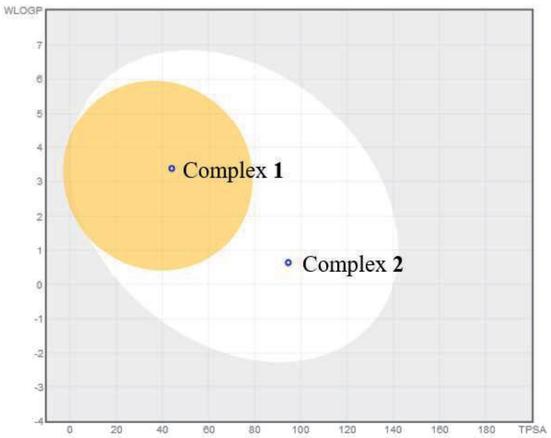

Figure S6. The Bolied–Egg diagram for the examined Ru complexes (generated by the SwissADME server accessed on 8 October 2024).

4. Supporting references

3. Rogala, P.; Czerwonka, G.; Michałkiewicz, S.; Hodorowicz, M.; Barszcz, B.; Jabłońska-Wawrzycka, A. Synthesis, structural characterization and antimicrobial evaluation of ruthenium complexes with heteroaromatic carboxylic acids. *Chem. Biodiversity* **2019**, *16*, e1900403. https://doi.org/10.1002/cbdv.201900403.

4. Czerwonka, G.; Gmiter, D.; Guzy, A.; Rogala, P.; Jabłońska-Wawrzycka, A.; Borkowski, A.; Cłapa, T.; Narożna, D.; Kowalczyk, P.; Syczewski, M.; et al. A benzimidazole-based ruthenium(IV) complex inhibits *Pseudomonas aeruginosa* biofilm formation by interacting with siderophores and the cell envelope, and inducing oxidative stress. *Biofouling* **2019**, *35*, 59–74. https://doi.org/10.1080/08927014.2018.1564818.

54. Velders, A.H.; Pazderski, L.; Ugozzoli, F.; Biagini-Cingi, M.; Manotti-Lanfredi, A.M.; Haasnoot, J.G.; Reedijk, J. Synthesis, characterization and crystal structure of *trans*-aquatrichlorobis (5,7-dimethyl[1,2,4]triazolo[1,5-a]pyrimidine-N^3)ruthenium(III) monohydrate. *Inorg. Chim. Acta* 1998, *273*, 259–265.

55. Blanc, R.; González-Casado, A.; Navalón, A.; Vílchez, J.L. On the estimate of blanks in differential pulse voltammetric techniques: application to detection limits evaluation as recommended by IUPAC. *Anal. Chim. Acta* 2000, *403*, 117–123. https://doi.org/10.1016/S0003-2670(99)00569-3.

56. Rigaku Oxford Diffraction, CrysAlisPro, Version. 1.171.36.20, Rigaku Oxford Diffraction, Yarnton, Oxfordshire, England, 2015.

57. Sheldrick, G.M. SHELXT – Integrated space-group and crystal-structure determination. *Acta Crystallogr. Sect. A Found. Adv.* 2015, *71*, 3–8. https://doi.org/10.1107/S2053273314026370

58. Sheldrick, G.M. Crystal structure refinement with SHELXL. *Acta Crystallogr. Sect. C Struct. Chem.* 2015, *C71*, 3–8. https://doi.org/10.1107/S2053229614024218 .

59. H. Putz, K. Brandenburg, Diamond - Crystal and Molecular Structure Visualization Crystal Impact, GbR, Kreuzherrenstr. 102, D-53227, Bonn, Germany.

60. Spackman, P.R.; Turner, M.J.; McKinnon, J.J.; Wolff, S.K.; Grimwood, D.J.; Jayatilaka, D.; Spackman, M.A. CrystalExplorer: a program for Hirshfeld surface analysis, visualization and quantitative analysis of molecular crystals. *J. Appl. Crystallogr.* 2021, *54*, 1006–1011. https://doi.org/10.1107/S1600576721002910.

61. Spackman, M.A.; Jayatilaka, D. Hirshfeld surface analysis. *CrystEngComm* 2009, *11*, 19–32. https://doi.org/10.1039/B818330A.

62. Spackman, M.A.; McKinnon, J.J. Fingerprinting intermolecular interactions in molecular crystals. *CrystEngComm* 2002, *4*, 378–392. https://doi.org/10.1039/B203191B.

63. Cullen, L.; Weiser, R.; Olszak, T.; Maldonado, R.F.; Moreira, A.S.; Slachmuylders, L.; Brackman, G.; Paunova-Krasteva, T.S.; Żarnowiec, P.; Czerwonka, G.; Reilly, J.; Drevinek, P.; et al. Phenotypic characterization of an international *Pseudomonas aeruginosa* reference panel: strains of cystic fibrosis (CF) origin show less in vivo virulence than non-CF strains. *Microbiology* 2015, *161*, 1961–1977. https://doi.org/10.1099/mic.0.000155.

64. Borkowski, A.; Gutowski, Ł.; Syczewski, M.; Cłapa, T.; Czerwonka, G. Adaptation of bacteria *Escherichia coli* in presence of quaternary ammonium ionic liquids. *Ecotoxicol. Environ. Saf.* 2018, *164*, 370–378. https://doi.org/10.1016/j.ecoenv.2018.08.048.

65. Berman, H.M.; Westbrook, J.; Feng, Z.; Gilliland, G.; Bhat, T.N.; Weissig, H.; Shindyalov, I.N.; Bourne, P.E. The Protein Data Bank. *Nucleic Acids Res.* 2000, *28*, 235–242. https://doi.org/10.1093/nar/28.1.235.

66. Schrödinger Release 2024-1: Maestro, Schrödinger, LLC, New York, NY 2021.

67. Macrae, C.F.; Sovago, I.; Cottrell, S.J.; Galek, P.T.A.; McCabe, P.; Pidcock, E.; Platings, M.; Shields, G.P.; Stevens, J.S.; Towler, M.; et al. Mercury 4.0: from visualization to analysis, design and prediction. *J. Appl. Crystallogr.* 2020, *53*, 226–235. https://doi.org/10.1107/S1600576719014092.

68. Verdonk, M.L.; Cole, J.C.; Hartshorn, M.J.; Murray, C.W.; Taylor, R.D. Improved protein–ligand docking using GOLD. *Proteins Struct. Funct. Bioinforma.* 2003, *52*, 609–623. https://doi.org/10.1002/prot.10465.

69. Sapundzhi, F.; Prodanova, K.; Lazarova, M. Survey of the scoring functions for protein-ligand docking. *AIP Conf. Proc.* 2019, *2172*, 100008. https://doi.org/10.1063/1.5133601.

70. The PyMOL Molecular Graphics System, Version 2.5 Schrödinger.

71. Salentin, S.; Schreiber, S.; Haupt, V.J.; Adasme, M.F.; Schroeder, M. PLIP: fully automated protein–ligand interaction profiler. *Nucleic Acids Res.* 2015, *43*, W443–W447. https://doi.org/10.1093/nar/gkv315.

72. Lakowicz, J.R. *Principles of Fluorescence Spectroscopy*; Springer US: Boston, MA, 2006; Vol. 1; ISBN 978-0-387-31278-1.

44. Lakowicz, J.R.; Weber, G. Quenching of protein fluorescence by oxygen. Detection of structural fluctuations in proteins on the nanosecond time scale. *Biochemistry* 1973, *12*, 4171–4179. https://doi.org/10.1021/bi00745a021.

73. Lineweaver, H.; Burk, D. The Determination of Enzyme Dissociation Constants. *J. Am. Chem. Soc.* 1934, *56*, 658–666. https://doi.org/10.1021/ja01318a036.
